# Transcription apparatus of the yeast virus-like elements: Architecture, function, and evolutionary origin

**DOI:** 10.1371/journal.ppat.1007377

**Published:** 2018-10-22

**Authors:** Michal Sýkora, Martin Pospíšek, Josef Novák, Silvia Mrvová, Libor Krásný, Václav Vopálenský

**Affiliations:** 1 Department of Genetics and Microbiology, Faculty of Science, Charles University, Prague, Czech Republic; 2 Institute of Microbiology, Academy of Sciences of the Czech Republic, Prague, Czech Republic; Plymouth University, UNITED KINGDOM

## Abstract

Extrachromosomal hereditary elements such as organelles, viruses, and plasmids are important for the cell fitness and survival. Their transcription is dependent on host cellular RNA polymerase (RNAP) or intrinsic RNAP encoded by these elements. The yeast *Kluyveromyces lactis* contains linear cytoplasmic DNA virus-like elements (VLEs, also known as linear plasmids) that bear genes encoding putative non-canonical two-subunit RNAP. Here, we describe the architecture and identify the evolutionary origin of this transcription machinery. We show that the two RNAP subunits interact *in vivo*, and this complex interacts with another two VLE-encoded proteins, namely the mRNA capping enzyme and a putative helicase. RNAP, mRNA capping enzyme and the helicase also interact with VLE-specific DNA *in vivo*. Further, we identify a promoter sequence element that causes 5′ mRNA polyadenylation of VLE-specific transcripts *via* RNAP slippage at the transcription initiation site, and structural elements that precede the termination sites. As a result, we present a first model of the yeast virus-like element transcription initiation and intrinsic termination. Finally, we demonstrate that VLE RNAP and its promoters display high similarity to poxviral RNAP and promoters of early poxviral genes, respectively, thereby pointing to their evolutionary origin.

## Author summary

Yeast cytoplasmic double-stranded DNA virus-like elements (VLEs, also known as linear plasmids) were widely investigated in the past but the topic was almost entirely abandoned, partly due to an inability to express VLE-encoded proteins using conventional expression systems. In this study, we re-opened investigation of these elements focused on considerably underexplored nucleus-independent transcription of *K*. *lactis* VLEs. Using systematic *in vivo* study, we were able to characterize composition of the previously hypothesized VLE-encoded transcription complex. Further, we identified new DNA and RNA elements that were directly connected with formation of VLE-specific mRNA ends, as demonstrated by mutagenesis of these elements and its effect on VLE transcription *in vivo*. Finally, our phylogenetic and sequence analysis of VLE-encoded non-canonical RNA polymerase and its promoters, respectively, suggested evolutionary relationship of VLE transcription machinery to much better explored transcription machinery of cytoplasmic double-stranded DNA viruses of the *Poxviridae* family.

## Introduction

Linear double-stranded DNA virus-like elements (VLEs) were found in the cytoplasm of several yeast species. Structural organization of these elements is quite uniform and they are often present as two or three differently sized DNA plasmids in yeast host cells [1]. Characteristic features of VLEs are terminal proteins covalently linked to the 5′ ends of their DNA, terminal inverted repeats, and their cytoplasmic localization [2–4]. Yeast linear plasmids of *Kluyveromyces lactis*, termed pGKL1 (or K1) and pGKL2 (or K2), have become a model system to study such DNA elements. These VLEs have compact genomes with occasional overlaps of open reading frames (ORFs) and a high AT content of ~74% [5, 6]. The presence of both pGKL plasmids in several *K*. *lactis* strains is associated with the extensively studied yeast killer phenotype [7].

Functions of protein products for most ORFs encoded by the pGKL plasmids were predicted using bioinformatics approaches and some of these proteins were characterized by biochemical and genetic analyses [1]. Both pGKL1 and pGKL2 encode their own DNA polymerase and a terminal protein, and it is assumed that the mechanism of their replication is similar to the replication of viruses of the *Adenoviridae* family or *Bacillus subtilis* bacteriophage φ29 [8]. Consequently, marked sequence similarities between viral enzymes and putative products of several linear plasmid ORFs with expected function in replication and transcription resulted in yeast linear DNA plasmids being called virus-like elements nowadays [9]. Hence, it is believed that these VLEs may have originated from endosymbiotic bacteria or a virus [10]. Nevertheless, the exact evolutionary origin of the yeast linear VLEs remains unclear.

Transcription of VLE-specific genes has been shown to be independent of mitochondrial [11] and nuclear RNA polymerases [8, 12–14], and probably utilizes a VLE-specific RNA polymerase (RNAP). Experiments with bacterial reporter and yeast nuclear genes fused with pGKL-derived sequences identified an upstream conserved sequence (5′-ATNTGA-3′) preceding each of the open reading frames. This upstream conserved sequence (UCS), usually located at a distance of 20–40 nucleotides prior to the start codon, is essential for cytoplasmatic transcription of the downstream located gene [15–17]. Sequences located farther upstream of the UCS element have been shown to have no effect on transcription [16]. The UCS element is highly conserved among all yeast VLEs and the UCS sequence derived from the *Pichia etchellsii* pPE1B element acts as a functional promoter when transplanted into the pGKL1 element [18]. Thus, the UCS element is a universal *cis*-acting component of the VLE-specific transcription system and it is essential for transcription initiation. After elongation, transcription then terminates after each gene because only monocistronic transcripts were revealed with Northern blot analyses of transcripts derived from ten pGKL-encoded ORFs [6, 12, 19–21]. This suggests the existence of a defined, yet unknown mechanism of transcription termination.

Unique RNAP subunits, and possibly also a putative helicase and the mRNA capping enzyme are the key elements of the VLE cytoplasmic transcription machinery. Protein products of *ORF6* (K2ORF6p; large subunit) and *ORF7* (K2ORF7p; small subunit) of the pGKL2 element should form a non-canonical RNAP. K2ORF6p was found to have a sequence similarity to three conserved regions of the two largest subunits (β and β′ in bacteria) of canonical multisubunit RNAPs [22]. Sequence similarity of K2ORF6p to the β and β′ subunit has recently been extended to 12 conserved regions shared by all bacterial, archaeal and eukaryotic RNAPs [23], and the predicted structure of this enzyme thus resembles a fusion of the β subunit with a portion of the β′ subunit. K2ORF7p was found to have sequence similarities to two conserved regions of the β′ subunit, which are usually located at the C-terminus of β′ [24].

The *ORF4* sequence of the pGKL2 element shows striking sequence similarity to viral helicases from the superfamily II of DEAD/H family helicases involved in transcription. The *K2ORF4* protein product (K2ORF4p) displays similarity with two Vaccinia virus helicases–(i) NPH-I, which is encoded by the *D11L* gene, and (ii) the small subunit of the heterodimeric Vaccinia virus early transcription factor (VETF) encoded by the *D6R* gene [22, 25]. NPH-I is known to provide the energy for elongation of transcription and for the release of RNA during transcription termination [26]. VETF functions as a transcription initiation factor that binds and bends the promoter region of early genes [27].

The protein product of *ORF3* (K2ORF3p) encoded by the pGKL2 element shows sequence similarity to the Vaccinia virus mRNA capping enzyme encoded by the *D1R* gene that consists of three domains responsible for the three enzymatic activities necessary to form the 5′ mRNA cap structure [28]. The methyltransferase activity of the D1 protein of the poxvirus Vaccinia is allosterically stimulated by heterodimerization with a smaller protein encoded by the *D12L* gene [29, 30]. The complex of D1 and D12 proteins is sometimes also referred to as the vaccinia termination factor (VTF) because, together with NPH-I, it also acts as a transcription termination factor of early genes [31]. Triphosphatase and guanylyltransferase activities of K2ORF3p have been already confirmed experimentally *in vitro* [32].

As reported previously, the *K2ORF3*, *K2ORF4*, *K2ORF6* and *K2ORF7* genes are indispensable for the maintenance of the pGKL elements in the cell [19, 20, 24, 32]. However, understanding of interactions of their protein products with each other and with VLE DNA in the cell is lacking, as well as understanding of DNA sequence elements required for VLE transcription initiation and termination.

Here, we present a systematic *in vivo* study focusing on the architecture of the transcription complex of the yeast VLEs. Moreover, we identify a new promoter DNA element which is associated with 5′ mRNA polyadenylation of most pGKL-encoded genes and we uncover a link between RNA stem loop structures and 3′ end formation of VLE-specific mRNAs *in vivo*. Further, we present an extensive phylogenetic analysis of amino acid sequences of VLE RNAP subunits. Finally, we provide a detailed sequence analysis of pGKL promoters. Collectively, these analyses strongly suggest that the VLE transcription machinery has origin close to poxviruses.

## Results

### VLE RNAP subunits, mRNA capping enzyme, and helicase associate *in vivo*

To start characterizing the transcription machinery of the yeast VLEs we first tested whether the VLE RNAP subunits (K2ORF6p, K2ORF7p), the mRNA capping enzyme (K2ORF3p), and the putative helicase (K2ORF4p) form a complex *in vivo*.

Initially, we tested interactions between K2ORF3p, K2ORF6p and K2ORF7p using a yeast two-hybrid system and its fluorescence variant called bimolecular fluorescence complementation but we failed to detect any interaction with either approach. This was most likely caused by the high AT content of VLE genes that was shown recently to impair their nuclear expression due to RNA fragmentation mediated by the polyadenylation machinery [33]. Therefore, we decided to prepare modified pGKL elements expressing the putative transcription machinery components containing various tags.

**Fig 1:**
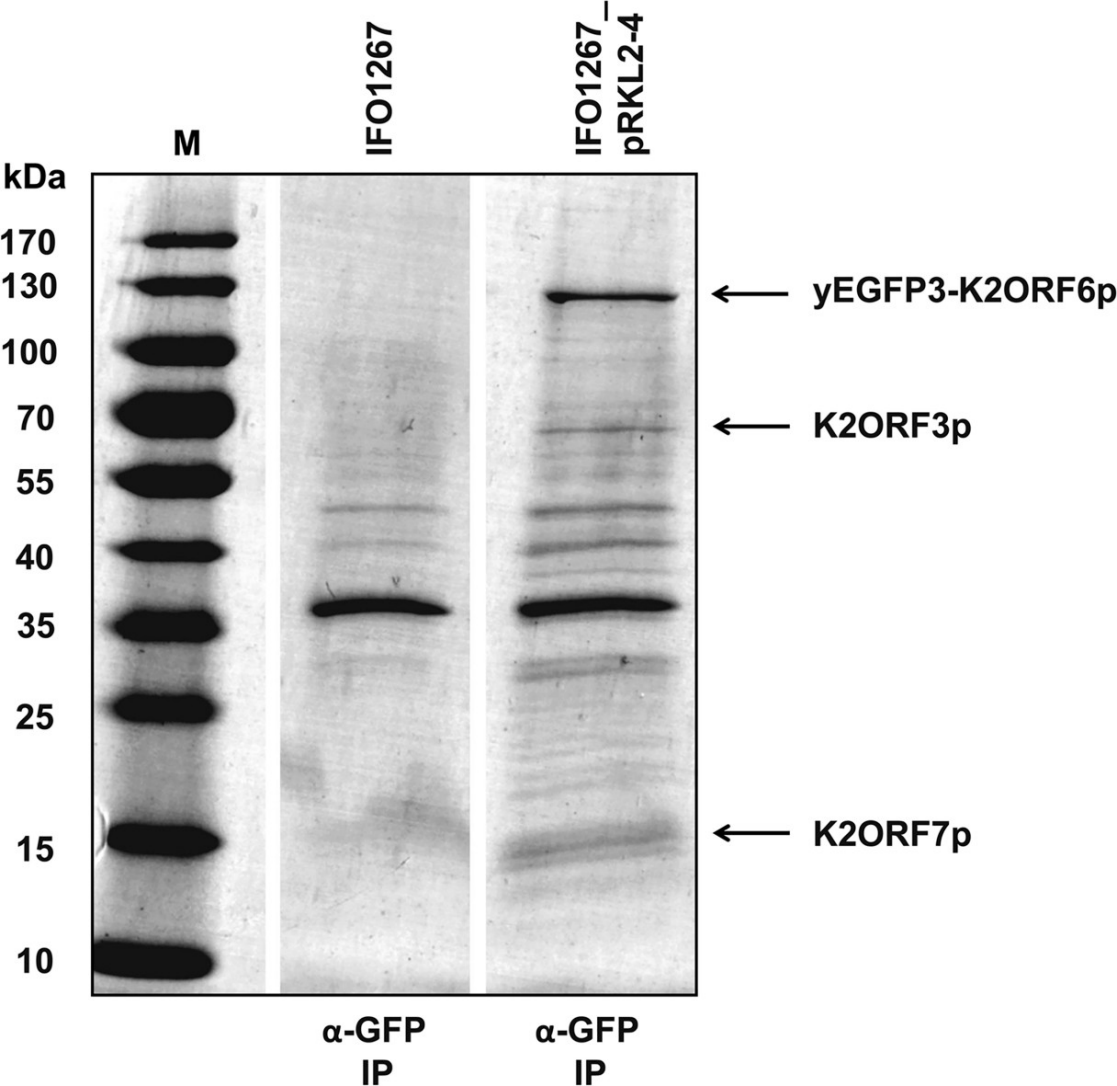
Identification of proteins associated with the large subunit of the VLE RNAP (K2ORF6p). The gel shows Coomassie stained proteins affinity-purified with GFP-Trap_A from strains IFO1267 (control) and IFO1267_pRKL2-4 (containing yEGFP3-K2ORF6p). Proteins identified by mass spectrometry are indicated with arrows on the right side, and also listed in Table 1. M, protein molecular mass marker (PageRuler Prestained Protein Ladder, Fermentas); the respective molecular mass values are indicated on the left side.

We prepared a strain encoding yeast enhanced green fluorescent protein 3 (yEGFP3) [34] that was fused to the N-terminus of the large RNAP subunit K2ORF6p (yEGFP3-K2ORF6p; strain IFO1267_pRKL2-4). We immunoprecipitated (IP) yEGFP3-K2ORF6p from this strain using GFP-Trap_A agarose beads that contain a monoclonal antibody against common GFP variants. As a control, we used the wt strain IFO1267 that had no modifications. Extensive washing was used to remove weakly bound proteins. The bound proteins were eluted, resolved on SDS-PAGE, and stained with Coomassie Brilliant Blue G-250 (Fig 1). Gel lanes or bands of interest were excised from the gel, and analysed by mass spectrometry (MS). As shown in Table 1, peptides corresponding to yEGFP3-K2ORF6p, K2ORF7p (RNAP small subunit) and K2ORF3p (mRNA capping enzyme) were detected whereas no such peptides were identified in the parallel-treated IFO1267 control sample.

**Table 1:** K2ORF6p-associated proteins.

Protein identified	MW (kDa)	Coverage	No. of peptides		Approach
			IFO1267_pRKL2-4	IFO1267	
yEGFP3-K2ORF6p	140.7	48%	170	0	whole lane analysis
K2ORF7p	15.5	53%	22	0	whole lane analysis
K2ORF3p	70.5	24%	13	-	band excision

IFO1267 (control) and IFO1267_pRKL2-4 (yEGFP3-K2ORF6p) cells were grown to late exponential phase. The cells were lysed and yEGFP3-K2ORF6p was affinity-purified using GFP-Trap agarose beads. Bound proteins were eluted and resolved by SDS-PAGE, stained with Coomassie Brilliant Blue G-250, and analysed by mass spectrometry. The proteins identified, their molecular weight (MW), unique coverage, and the number of the detected peptides from both strains is listed.

Then, to verify the interaction between the large RNAP subunit and the mRNA capping enzyme we decided to perform immunoprecipitation using tagged K2ORF3p as the bait. Hence, we prepared a strain encoding yEGFP3 fused to the C-terminus of K2ORF3p (IFO1267_pRKL2-11 strain). Interestingly, selective cultivation of clones after transformation led to a loss of the pGKL1 element (S1 Fig). Immunoprecipitation using GFP-Trap_A agarose beads and subsequent MS analysis revealed peptides corresponding to K2ORF6p, K2ORF7p, K2ORF4p, and K2ORF3p-yEGFP3 (S2 Fig).

**Fig 2:**
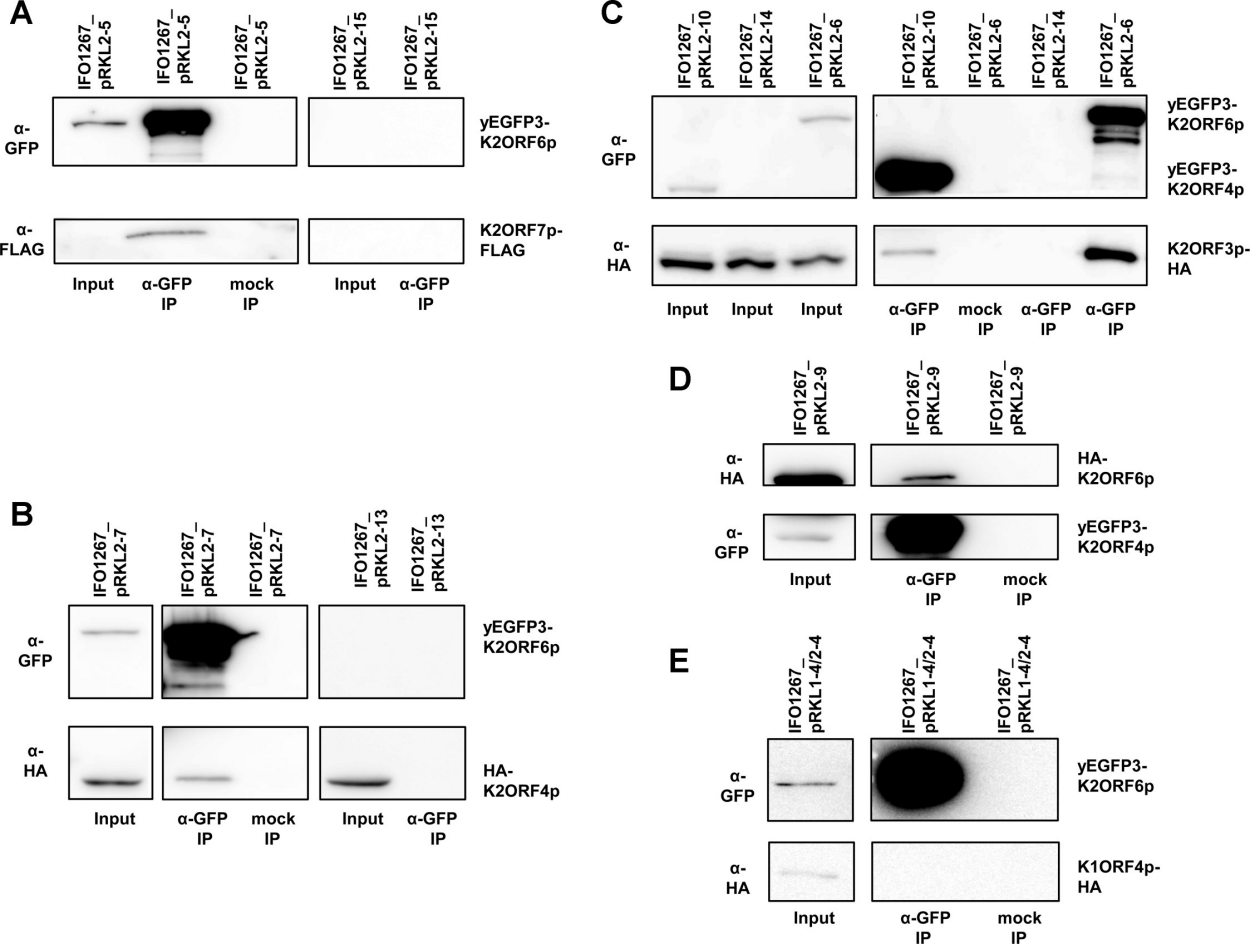
Interactions between RNAP subunits, mRNA capping enzyme, and putative helicase of the yeast VLEs. (A) Western blot of immunoprecipitated (α-GFP IP) and mock immunoprecipitated (mock IP) proteins from IFO1267_pRKL2-5 (yEGFP3-K2ORF6p, K2ORF7p-FLAG) or control IFO1267_pRKL2-15 cells (K2ORF7p-FLAG), respectively. The strains used are indicated above the lanes. The antibodies used for Western blot are indicated on the left hand side of the strips. The proteins detected are indicated on the right hand side of the strips. Positions of the identified proteins corresponded with theoretical molecular weight of the full length recombinant proteins, as determined by positions of the protein mass markers. Input represented approximately 1/100 of the sample and IP represented approximately 1/2 of the sample in this and the other immunoprecipitation experiments. Mock immunoprecipitations in all experiments were done using empty agarose beads. The same experimental scheme is used throughout this figure. (B) Western blot analysis of immunoprecipitations from lysates from IFO1267_pRKL2-7 (yEGFP3-K2ORF6p, HA-K2ORF4p) and control IFO1267_pRKL2-13 (HA-K2ORF4p) cells (indicated above the lanes). The (α-GFP) and anti-HA (α-HA) antibodies used are indicated on the left hand side; the detected proteins on the right hand side. (C) Western blot analysis of immunoprecipitations from lysates from strains IFO1267_pRKL2-6 (yEGFP3-K2ORF6p, K2ORF3p-HA), IFO1267_pRKL2-10 (yEGFP3-K2ORF4p, K2ORF3p-HA), and IFO1267_pRKL2-14 (control). (D) Western blot analysis of immunoprecipitations from lysates from IFO1267_pRKL2-9 cells (yEGFP3-K2ORF4p, HA-K2ORF6p). (E) Western blot analysis of immunoprecipitations from lysates from IFO1267_pRKL1-4/2-4 cells (yEGFP3-K2ORF6p, K1ORF4p-HA).

To further validate the results, we prepared several strains containing combinations of these proteins with various tags: (i) strain IFO1267_pRKL2-5 where yEGFP3-K2ORF6p was co-expressed together with K2ORF7p-FLAG, and control strain IFO1267_pRKL2-15 that expressed only one tagged protein—K2ORF7p-FLAG; (ii) strain IFO1267_pRKL2-6 co-expressing yEGFP3-K2ORF6p and K2ORF3p-HA, and control strain IFO1267_pRKL2-14 expressing only K2ORF3p-HA. The results of IP experiments followed with Western blotting clearly showed specific interactions between these proteins (Fig 2A and 2C).

Thus far, we had detected K2ORF3p, K2ORF6p, and K2ORF7p to be associated *in vivo*. For the fourth protein, the putative helicase K2ORF4p, the MS results suggesting it as part of the complex were not convincing due to low protein coverage (S2 Fig). To address whether it does, although perhaps weakly, interact with these proteins, we prepared a strain expressing HA-K2ORF4p together with yEGFP3-K2ORF6p (IFO1267_pRKL2-7), and a control strain expressing HA-K2ORF4p only (IFO1267_pRKL2-13). After IP and Western blotting we found HA-K2ORF4p to associate with yEGFP3-K2ORF6p (Fig 2B). The results clearly showed that the putative helicase was specifically associated with the large RNAP subunit (or, was present in a complex containing this subunit) because yEGFP3-K2ORF6p and HA-K2ORF4p were not bound to the empty agarose beads, and HA-K2ORF4p alone did not bind to the GFP-Trap antibody (Fig 2B).

Because the association of the putative helicase with the large RNAP subunit seemed rather weak, we decided to perform reciprocal immunoprecipitation. Further, we also tested whether the mRNA capping enzyme associated with the putative helicase. Strains expressing (i) yEGFP3-K2ORF4p together with HA-K2ORF6p (IFO1267_pRKL2-9), and (ii) yEGFP3-K2ORF4p together with K2ORF3p-HA (IFO1267_pRKL2-10) were prepared. With the first combination we confirmed that HA-K2ORF6p associated with yEGFP3-K2ORF4p (Fig 2D). With the second combination we found that K2ORF3p-HA associated with yEGFP3-K2ORF4p (Fig 2C). The detected interactions were specific because yEGFP3-K2ORF4p and HA-K2ORF6p were not bound to the empty agarose beads and K2ORF3p-HA alone did not bind to the GFP-Trap antibody (Fig 2C and 2D).

As an additional control to demonstrate that the observed interactions were specific, we used another pGKL-encoded protein with a function unrelated to transcription. We selected K1ORF4p, a subunit of the toxin responsible for VLE-associated killer yeast phenotype [35]. A strain co-expressing yEGFP3-K2ORF6p together with K1ORF4p-HA (IFO1267_pRKL1-4/2-4) was prepared. We found that K1ORF4p-HA was not associated with yEGFP3-K2ORF6p (Fig 2E).

Finally, we wanted to know whether the association of the putative helicase and mRNA capping enzyme with the large RNAP subunit was dependent on nucleic acids. We prepared lysates from IFO1267_pRKL2-6 (yEGFP3-K2ORF6p, K2ORF3p-HA) and IFO1267_pRKL2-7 (yEGFP3-K2ORF6p, HA-K2ORF4p) strains. The lysates were incubated with GFP-Trap_A beads and, after washing, the beads were split into two parts which were treated or mock-treated with Benzonase Nuclease to digest DNA and RNA. Then, the beads were again extensively washed and the bound proteins were eluted. Subsequent Western blot analysis revealed both K2ORF3p-HA and HA-K2ORF4p to associate with yEGFP3-K2ORF6p regardless of the presence or absence of nucleic acids which was confirmed by PCR (S3 Fig).

Taken together, the immunoprecipitation, mass spectrometry, and Western blot results demonstrated the existence of the hypothesized VLE-specific transcription complex because K2ORF3p, K2ORF4p, K2ORF6p, and K2ORF7p were specifically associated *in vivo*. This association was independent of nucleic acids. Finally, K2ORF3p, K2ORF6p, and K2ORF7p appeared to form a core transcription complex with relatively strong mutual interactions to which K2ORF4p bound relatively weakly.

### RNAP, mRNA capping enzyme, and helicase are associated with VLE-specific DNA *in vivo*

**Fig 3:**
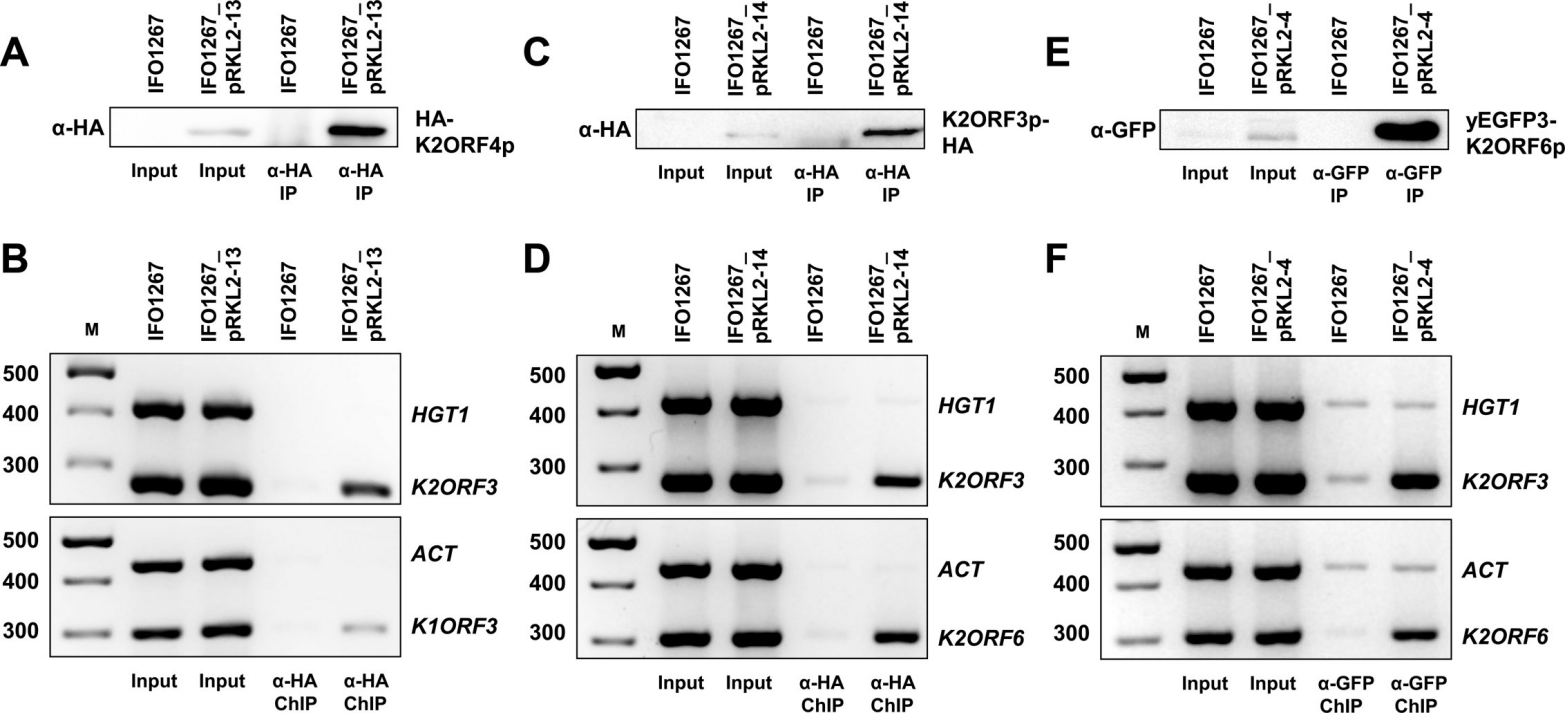
Physical association of the putative helicase (K2ORF4p), mRNA capping enzyme (K2ORF3p), and the large RNAP subunit (K2ORF6p) of the yeast VLEs with VLE-specific DNA. (A) Western blot of HA-K2ORF4p that was affinity-purified from lysates of IFO1267_pRKL2-13 (HA-K2ORF4p) and IFO1267 (control) cells. The strains used are indicated above the lanes. The antibody used is indicated on the left hand side of the strip. The protein detected is indicated on the right hand side of the strip. (B) PCR analysis of the presence of chromosomal (*ACT*, *HGT1*) or VLE (*K1ORF3*, *K2ORF3*) DNA in chromatin immunoprecipitated using anti-HA HA-7 agarose from IFO1267_pRKL2-13 (HA-K2ORF4p) and IFO1267 (control) cells. Samples of individually performed gene-specific PCRs were analysed in 2.5% agarose gel stained with ethidium bromide. The identity of the bands (genes) is indicated on the right. M, DNA molecular mass marker (GeneRuler 100 bp Plus DNA Ladder, Fermentas). The respective values are indicated on the left. (C) Western blot of K2ORF3p-HA that was affinity-purified from lysates of IFO1267_pRKL2-14 (K2ORF3p-HA) and IFO1267 (control) cells. (D) PCR analysis of the presence of chromosomal (*ACT*, *HGT1*) or VLE (*K2ORF3*, *K2ORF6*) DNA in chromatin immunoprecipitated using anti-HA HA-7 agarose from IFO1267_pRKL2-14 and IFO1267 cells. (E) Western blot of yEGFP3-K2ORF6p that was affinity-purified from lysates of IFO1267_pRKL2-4 (yEGFP3-K2ORF6p) and IFO1267 (control) cells. (F) PCR analysis of the presence of chromosomal (*ACT*, *HGT1*) or VLE (*K2ORF3*, *K2ORF6*) DNA in chromatin immunoprecipitated using GFP-Trap agarose beads from IFO1267_pRKL2-4 and IFO1267 cells.

Previous results confirmed the existence of the hypothesized VLE transcription complex *in vivo*. It is believed that this transcription complex is VLE-specific [22]. Therefore, it should possibly interact only with the VLE DNA *in vivo*, which was never formally tested. Hence, we performed *in vivo* chromatin immunoprecipitation. We used the IFO1267_pRKL2-13 strain expressing HA-K2ORF4p, IFO1267_pRKL2-14 strain expressing K2ORF3p-HA, IFO1267_pRKL2-4 strain expressing yEGFP3-K2ORF6p, and the IFO1267 control strain. First, we verified that the mouse monoclonal anti-HA HA-7 agarose efficiently immunoprecipitated HA-K2ORF4p and K2ORF3p-HA (Fig 3A and 3C), and that the GFP-Trap_A agarose beads efficiently immunoprecipitated yEGFP3-K2ORF6p (Fig 3E). Then, we performed chromatin immunoprecipitation of HA-K2ORF4p, K2ORF3p-HA, and yEGFP3-K2ORF6p from formaldehyde cross-linked cells. The immunoprecipitated and input DNAs were used as templates for subsequent PCR analysis using primers designed to detect chromosomal or VLE DNA. We used primers specific for *K*. *lactis* actin (*ACT*) and high-affinity glucose transporter (*HGT1*) genes as markers of chromosomal DNA, and toxin immunity (*K1ORF3*), mRNA capping enzyme (*K2ORF3*), and large RNAP subunit (*K2ORF6*) genes as markers of pGKL elements. We found, that HA-K2ORF4p, K2ORF3p-HA, and yEGFP3-K2ORF6p were specifically associated with pGKL elements and not with chromosomal DNA (Fig 3B, 3D and 3F). These results were also confirmed by semiquantitative real-time PCR.

We concluded that the mRNA capping enzyme, and previously uncharacterized RNAP and helicase were associated with VLE-specific DNA *in vivo*, which further supports involvement of these proteins in transcription of VLEs.

### Slippage of RNAP at the initiation site results in 5′ polyadenylation of VLE mRNAs

Next, we wished to characterize transcription initiation of the VLE genes. Our previous 5′ RACE-PCR experiments had revealed 5′ cap structures on the VLE-specific mRNAs, likely synthetized by VLE-encoded K2ORF3p mRNA capping enzyme, and also the presence of non-templated 5′ poly(A) leaders of heterogeneous lengths in mRNAs of 12 pGKL genes except for *K2ORF2*, *K2ORF3* and *K2ORF8* [36]. Interestingly, heterogeneous 5′ poly(A) leaders are a known feature of poxviral intermediate and late transcripts [37, 38]. It was shown that the 5′ poly(A) leader was produced by slippage of Vaccinia virus RNAP on three consecutive thymidine residues in the template strand at the initiator region (INR) where transcription starts both *in vivo* and *in vitro* [39, 40].

**Fig 4:**
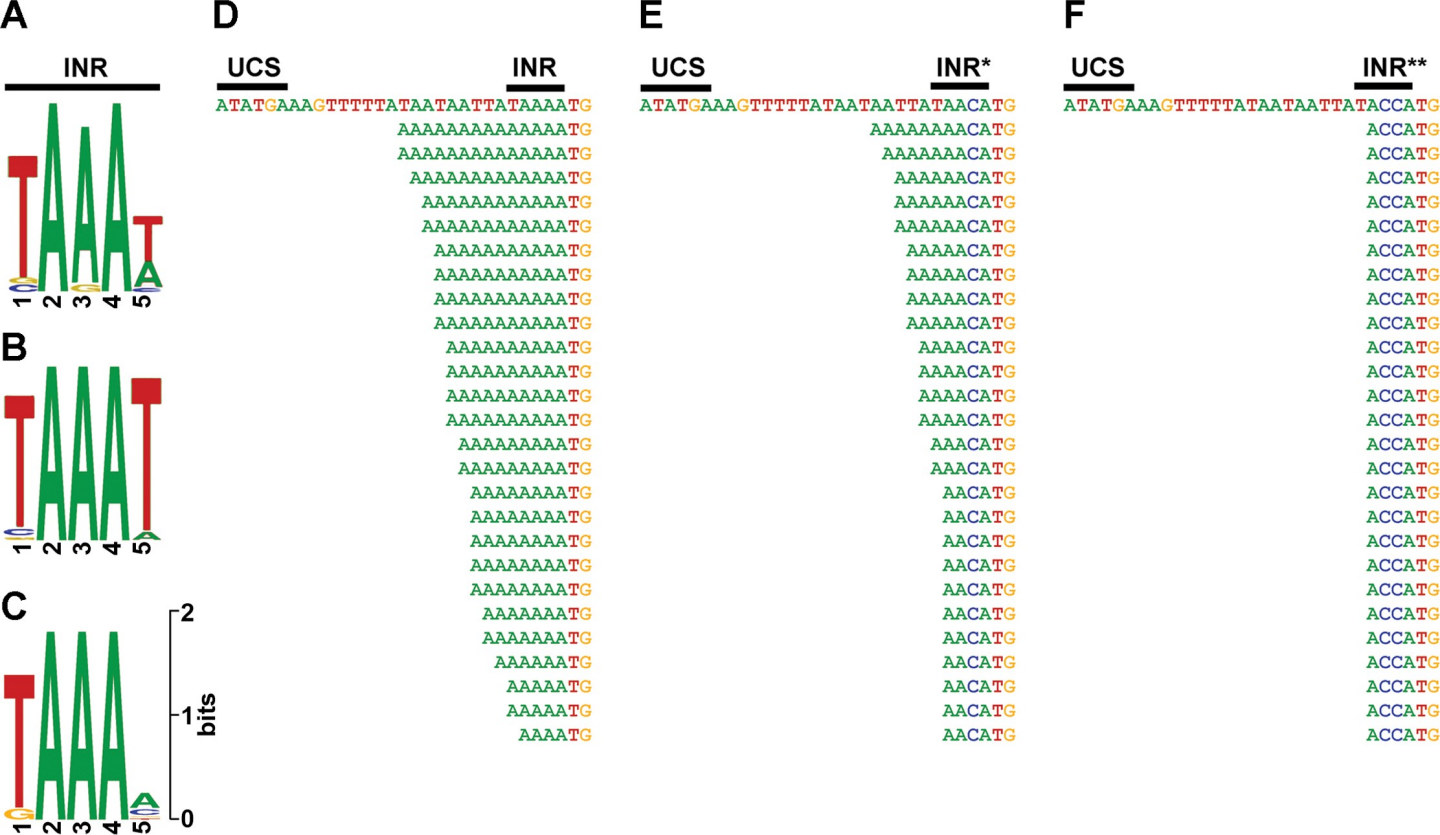
Promoters of yeast VLEs contain initiator region (INR) responsible for non-templated 5′ polyadenylation of mRNAs. (A) Sequence logo of the initiator region in promoter sequences of Vaccinia virus intermediate genes [41]. (B) Sequence logo of the initiator region in promoter sequences of Vaccinia virus late genes [41]. (C) Sequence logo of the putative INR identified in promoters of 12 ORFs encoded by pGKL elements. (D) 5′ RACE-PCR analysis of the *G418*^*R*^ gene from the IFO1267_pRKL1-1 strain. In this and the following panels, the upper sequence corresponds to the template (plasmid) DNA and the UCS is indicated; sequences situated below represent individual sequenced cDNA clones (the 5′ untranslated region is shown in full till the translation start codon, ATG). Guanosine residues corresponding to the original 5′ mRNA caps which were present in some of the cDNA clones are omitted in this representation for clarity. (E) 5′ RACE-PCR analysis of the *G418*^*R*^ gene from the IFO1267_pRKL1-2 strain bearing a promoter mutation in the putative INR reducing the number of consecutive adenosine residues in the template. (F) 5′ RACE-PCR analysis of the *G418*^*R*^ gene from the IFO1267_pRKL1-3 strain bearing promoter mutations in the putative INR abolishing consecutive adenosine residues in the template.

Fig 4A and 4B show the sequence logo of the INR consensus motif (TAAAT) of Vaccinia virus intermediate and late genes, respectively [41]. Interestingly, we were able to locate a similar INR-like consensus sequence (TAAAN) in promoters of all 12 pGKL genes whose transcripts were 5′ polyadenylated (Fig 4C). For the transcription start site (TSS) annotation, the first adenosine residue of the motif was considered to encode the initiating nucleoside triphosphate.

Subsequently, we tested whether the putative INR was responsible for the 5′ end polyadenylation of the pGKL-derived transcripts. We prepared three *K*. *lactis* strains with modified pGKL1 elements encoding the G418 resistance marker under the control of the K1UCR2 promoter. We prepared three variants of the K1UCR2 promoter that differed in the INR sequence: (i) TAAAA (wt; strain IFO1267_pRKL1-1); (ii) TAACA (strain IFO1267_pRKL1-2); and (iii) TACCA (strain IFO1267_pRKL1-3). Then, we purified total RNA from the three strains, prepared cDNA, and performed 5′ RACE-PCR to determine the 5′ end sequences. The results showed that the 5′ poly(A) leader was present when the K1UCR2 sequence contained the putative wt INR (TAAAA INR) (Fig 4D). When TAACA INR* was used, the length of the 5′ end poly(A) was significantly reduced (Fig 4E). When TACCA INR** was used, the poly(A) leader disappeared altogether (Fig 4F).

Therefore, we concluded that slippage of VLE RNAP at the initiation site was the mechanism responsible for 5′ polyadenylation of the transcripts. Moreover, the identified INR sequence constituted an independent DNA element, not influenced by the sequence of the gene because the pattern of the sequenced 5′ RACE-PCR clones for *K1ORF2* transcripts was the same as for *G418*^*R*^ transcripts produced from the K1UCR2 with the wt INR [36].

### RNA stem loop structures influence 3′ end formation of VLE-specific mRNAs *in vivo*

Our previous 3′ RACE-PCR experiments had revealed the absence of 3′ poly(A) tails in mRNAs of all 15 pGKL ORFs [36]. To shed light on the mode of transcription termination of the VLEs, we tried to identify sequence/secondary structure elements/signals near the 3′ termini. First, we searched for sequence motifs within the last 150 nt of each transcript that would be shared among the 15 pGKL ORFs but we detected none. Second, we searched for secondary structure motifs using the RNAstructure Server [42]. We identified putative RNA stem loop structures close to the experimentally determined 3′ ends of cDNA (S4 Fig). The putative RNA stem loops were typically in the vicinity of the respective ORF’s stop codon with the median distance of 26 nt, and Gibbs free energy of −7.5 kcal/mol.

**Fig 5:**
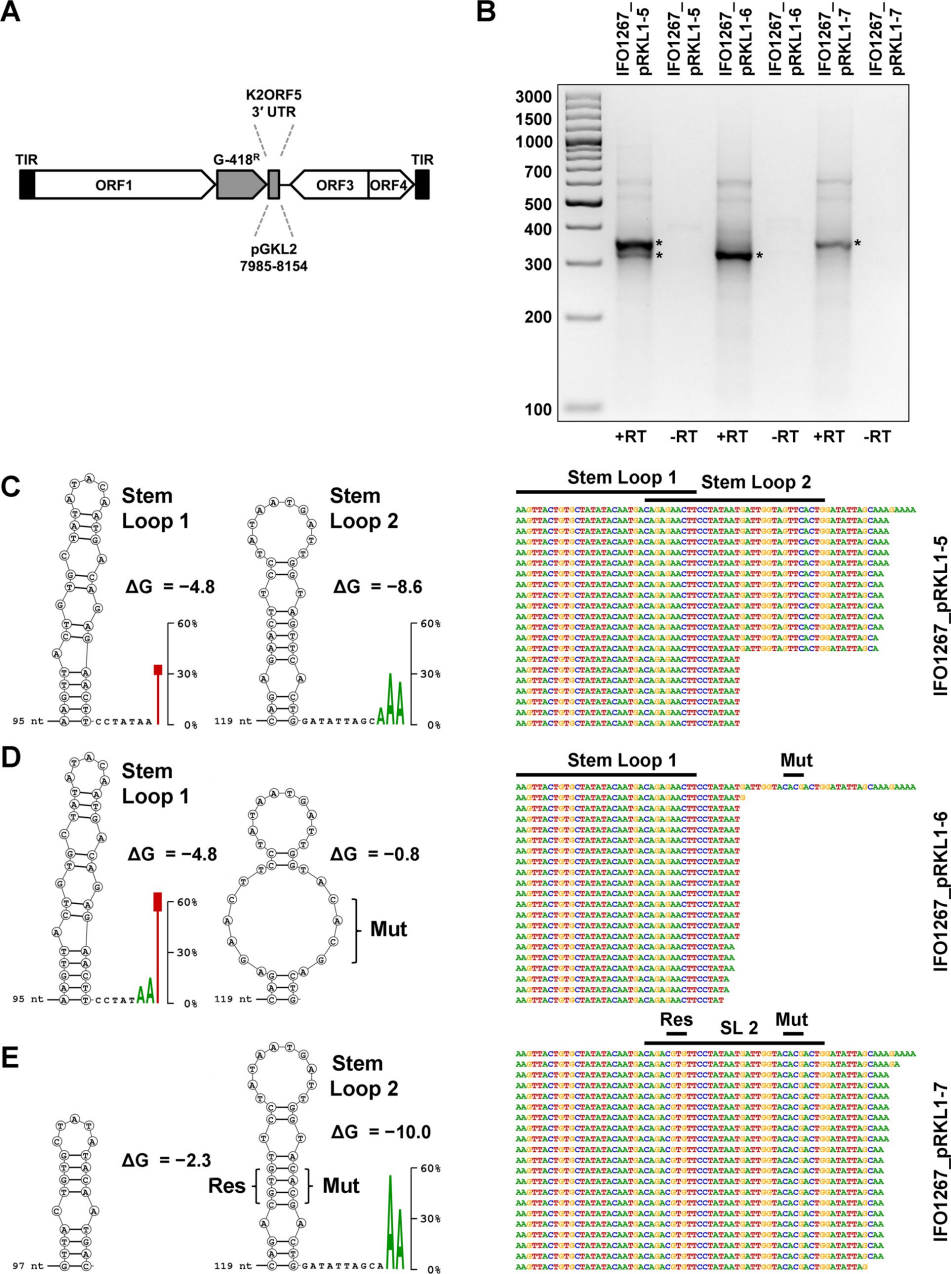
RNA stem loop structures influence the 3′ end formation of VLE-specific mRNAs *in vivo*. (A) Schematic representation of recombinant pGKL1 elements where the *G418*^*R*^ gene is followed by the coding sequence of wild-type (pRKL1-5) or modified (pRKL1-6, pRKL1-7) 3′ untranslated region of the *K2ORF5* gene. TIR—terminal inverted repeat. (B) 3′ RACE-PCR analysis of individual mRNAs corresponding to the *G418*^*R*^ gene expressed from modified pGKL1 elements. Samples were analyzed in 3.0% agarose gel stained by ethidium bromide. The strains used to purify the RNA are indicated above the lanes. M, DNA molecular mass marker (GeneRuler 100 bp Plus DNA Ladder, Fermentas). The respective values are indicated on the left. Specific products that were cloned to the pCR4-TOPO vector and used for sequencing are labelled with asterisks. Reverse transcription was carried out in the presence (+RT) and absence (-RT) of reverse transcriptase. (C) 3′ RACE-PCR analysis of the *G418*^*R*^ gene from IFO1267_pRKL1-5 strain. In this and the following panels, the upper sequences on the right correspond to the template (plasmid) DNA; sequences situated below represent 3′ end regions of individual sequenced cDNA clones. Positions of the putative RNA stem loops are indicated above the sequences. Predicted RNA stem loops are displayed as cDNA nucleotide letters in circles on the left and the values of Gibbs free energy (ΔG) in kcal/mol are displayed for each structure. Stem loop distances from the gene stop codon are shown as numbers of nucleotides. The last few 3**′** end nucleotides of the experimentally determined 3′ ends of cDNA are shown as letters enlarged proportionally to their occurrence (in %) in the sequenced clones in the case when these nucleotides were detected in at least two independent clones. (D) 3′ RACE-PCR analysis of the *G418*^*R*^ gene from the IFO1267_pRKL1-6 strain. Mut, the mutated stem loop. (E) 3′ RACE-PCR analysis of the *G418*^*R*^ gene from IFO1267_pRKL1-7 strain. Mut, the mutated stem loop; Res, the rescued stem loop.

Hence, we tested, whether the predicted RNA stem loop structures influenced the 3′ mRNA end formation. Because pGKL elements contain almost no intergenic regions, the putative RNA stem loops are localized in the coding sequences of adjacent ORFs or within the terminal inverted repeats. This means that their sequences cannot be subjected to mutagenesis without the possibility of altering VLE functions. Therefore, we prepared a *K*. *lactis* strain with a modified pGKL1 element encoding the G418 resistance marker under control of K1UCR2, followed by the 3′ UTR of the *K2ORF5* gene (strain IFO1267_pRKL1-5; Fig 5A). The distal part of the *K2ORF5* 3′ UTR contained two putative partially overlapping RNA stem loops termed Stem loop 1 and 2 (S4I Fig). 3′ RACE-PCR experiments revealed a transcription termination pattern that could be attributed to the presence of both Stem loop 1 and 2 (Fig 5B and 5C). Next, using the same promoter-gene-3′ UTR arrangement, we prepared a strain with 4 nucleotide mutations destabilizing the base pairing in the middle of the putative Stem loop 2 (strain IFO1267_pRKL1-6). For this construct, we detected a transcription termination pattern that could be attributed to the presence of only Stem loop 1 (Fig 5B and 5D). Subsequently, we prepared a strain (IFO1267_pRKL1-7) where we changed the sequence but not the base-pairing of 4 nucleotides within the Stem loop 2. 3′ RACE-PCR experiments revealed a transcription termination pattern that could be attributed to the presence of the rescued Stem loop 2 (Fig 5B and 5E). Note that the rescue mutations distinctly altered the length and the Gibbs free energy (destabilizing Stem loop 1) of the overlapping Stem loop 1 and this was likely the reason why transcription termination from the Stem loop 1 was not detected in the IFO1267_pRKL1-7 strain (Fig 5B and 5E).

Finally, we determined whether promoter sequences or coding sequences of a gene could affect the 3′ mRNA end formation. We used *K2ORF5*, G418, and hygromycin B resistance genes under the control of K2UCR5, K1UCR2 and K1UCR3, respectively, located on the pGKL2 element. Downstream of the coding sequence of each of these genes we positioned the 3′ untranslated region (UTR) of the *K2ORF5* gene. We purified total RNA from the respective *K*. *lactis* strains, prepared cDNA, and performed 3′ RACE-PCR experiments. Although the coding sequences of the aforementioned genes differed both in length and AT content, the pattern of their 3′ termini was highly similar (S4 and S5 Figs).

We concluded that RNA stem loop structures were essential for the 3′ end formation of VLE-specific mRNAs *in vivo*, presumably acting as factor-independent intrinsic terminators. Moreover, this termination was independent of the promoter and the gene used both with respect to its sequence and length.

### VLE-specific RNA polymerase has unique architecture

To facilitate interpretation of the experimental data we created a 3D model of the pGKL-specific RNAP. This was feasible due to sequence similarity between parts of K2ORF6p, K2ORF7p and conserved regions of the canonical multisubunit RNAPs [20, 23]. The structural models of the two subunits covered 92.7% of the K2ORF6p sequence and 62.1% of the K2ORF7p sequence, respectively.

Superimposition of the models over the *S*. *cerevisiae* RNAP II elongation complex (PDB ID: 2NVQ) using DaliLite v. 3 showed that all known regions of K2ORF6p and K2ORF7p with sequence similarity to conserved regions of the canonical RNAPs were modelled accordingly. Interestingly, parts of K2ORF6p were modelled by βa1, βa6, βa13, and βa16 conserved regions, which were not detected previously to be present in K2ORF6p. We verified the proper model:template alignment of these regions by manually constructed sequence alignments with other canonical RNAPs and these alignments indeed showed sequence similarities between K2ORF6p and the aforementioned regions (S6 Fig).

**Fig 6:**
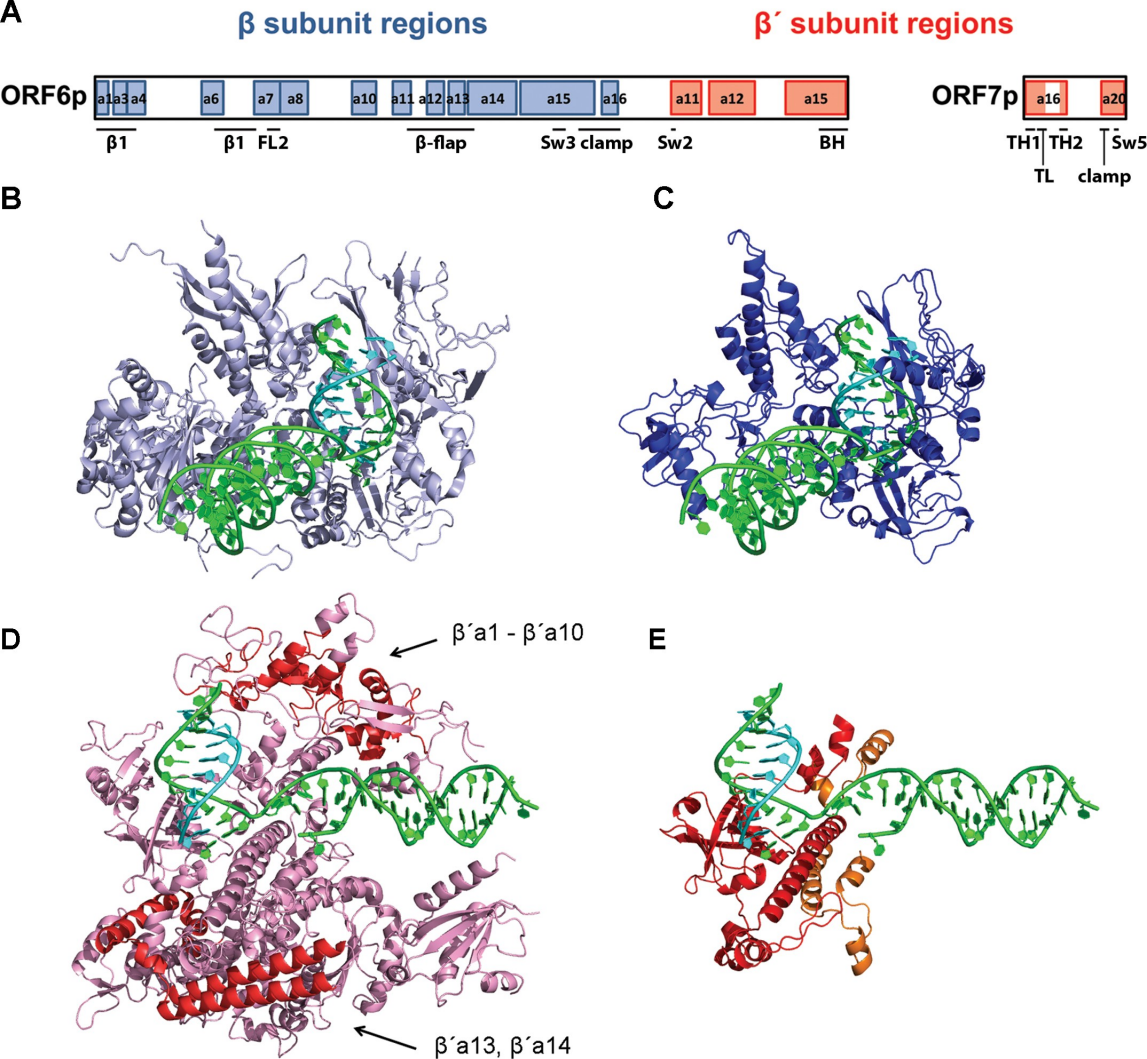
Predicted 3D structure of yeast VLE non-canonical RNAP encoded by the *ORF6* and *ORF7* genes. (A) Schematic representation of the primary sequence of pGKL-encoded RNAP showing similarity to conserved regions of the catalytic subunits of canonical multisubunit RNAPs. β subunit conserved regions (blue) and β′ subunit conserved regions (red) present in K2ORF6p and K2ORF7p are drawn to scale. Conserved regions are named according to ref. [46]. Sequence alignments for newly detected similarity of *ORF6* protein products to βa1, βa6, βa13 and βa16 conserved regions are provided in S6 Fig. BH, Bridge helix; FL2, Fork loop 2; Sw2, Switch 2; Sw3, Switch 3; Sw5, Switch 5; TH1, Trigger helix 1; TH2, Trigger helix 2; TL, Trigger loop. (B) 3D crystal structure of *Saccharomyces cerevisiae* RNA polymerase II elongation complex showing the Rpb2 subunit (β subunit homolog, light blue), DNA (green) and RNA (cyan). This figure is based on 2NVQ. (C) 3D model of pGKL RNAP showing K2ORF6p residues 1–693 (β subunit homolog, blue), DNA (green) and RNA (cyan). Nucleic acids in this as well as the following structures are based on 2NVQ. The structural similarity between the pGKL RNAP β subunit and RNAP II Rpb2 as calculated by DaliLite v. 3 is a root-mean-square deviation (RMSD) of 3.9 Å over 655 aligned Cα positions, 23% sequence identity, and the TM-score 0.87. (D) 3D crystal structure of *Saccharomyces cerevisiae* RNAP II elongation complex showing the Rpb1 subunit (β′ subunit homolog, pink), DNA (green) and RNA (cyan). Arrows indicate β′ regions shared by multisubunit RNAPs (red) that are clearly missing in the VLE RNAP. This figure is based on 2NVQ. (E) 3D model of pGKL RNAP showing K2ORF6p residues 754–882 and 894–974 (β′ subunit homolog, red), K2ORF7p residues 1–52 and 103–132 (β′ subunit homolog, orange), DNA (green) and RNA (cyan). The structural similarity between pGKL RNAP β′ subunit and RNAP II Rpb1 as calculated by DaliLite v. 3 is a RMSD of 2.3 Å over 123 aligned Cα positions, 31% sequence identity, and the TM-score 0.89 for K2ORF6p residues 754–882; RMSD of 1.4 Å over 80 aligned Cα positions, 21% sequence identity, and the TM-score 0.86 for K2ORF6p residues 894–974; RMSD of 2.9 Å over 43 aligned Cα positions, 23% sequence identity, and the TM-score 0.57 for K2ORF7p residues 1–52; RMSD of 1.3 Å over 29 aligned Cα positions, 13% sequence identity, and the TM-score 0.56 for K2ORF7p residues 103–132. For details concerning structure modelling see Materials and methods.

The overall distribution of the conserved regions within K2ORF6p and K2ORF7p is depicted in Fig 6A. It should be noted that K2ORF6p displayed a unique fusion between β and β′ subunit conserved regions, which is not known to be present in any other canonical or non-canonical RNAP. This fusion seemed to be essential for the maintenance of pGKL elements in yeast—a VLE where we divided *K2ORF6* into two genes, based on their homology to β and β′, was unable to substitute wt pGKL2 in the cell. On the other hand, a division of the β′ subunit (between β′a15 and β′a16) into two polypeptides, similarly as in K2ORF6p and K2ORF7p, is present in RNAPs of some *Archaeal* species [43].

Fig 6B and 6C show the *S*. *cerevisiae* RNAP II Rpb2 subunit (β subunit homolog) and the K2ORF6p model, respectively. It is clear that almost all conserved β subunit regions are present in K2ORF6p, and only the spacing between them is shorter. Fig 6D and 6E show the *S*. *cerevisiae* RNAP II Rpb1 subunit (β′ subunit homolog) and relevant homologous portions of K2ORF6p/K2ORF7p subunits, respectively. Remarkably, more than half of the β′ subunit conserved regions, such as most of the clamp domain (β′a1 - β′a10 regions) and secondary-channel rim helices (β′a13, β′a14 regions), are missing in VLE RNAP (Fig 6D).

We concluded that the VLE RNAP displayed a unique and novel architecture.

### VLE-specific RNA polymerase has a viral origin

An extensive phylogenetic analysis of yeast VLE RNAPs has not been performed yet. Earlier analyses focused only on conserved regions β′a11 and β′a12 of K2ORF6p. These studies established that VLE RNAP belongs to multisubunit RNAPs rather than to single-subunit RNAPs encoded by mitochondrial linear plasmids of fungi and plants [10, 44]. Hence, it is believed that the *ORF6* and *ORF7* genes of VLEs were derived from eukaryotic multisubunit RNAP genes of ancestral host cells [1, 23] or, alternatively, that the *ORF6* gene is an ancient representative of multisubunit RNAP diversification from times when β and β′ constituted a single protein [45].

We performed a detailed phylogenetic analysis to delve deep into the evolutionary past of yeast VLEs. We used sequences of β′a11-β′a12, β′a15-β′a16 and β′a20 conserved regions of ORF6 and ORF7 proteins from all sequenced yeast VLEs. Alignment containing these conserved regions was then combined with the published alignment of β′ subunit conserved regions of canonical multisubunit RNAPs [46] and was used to construct a maximum likelihood phylogenetic tree.

**Fig 7:**
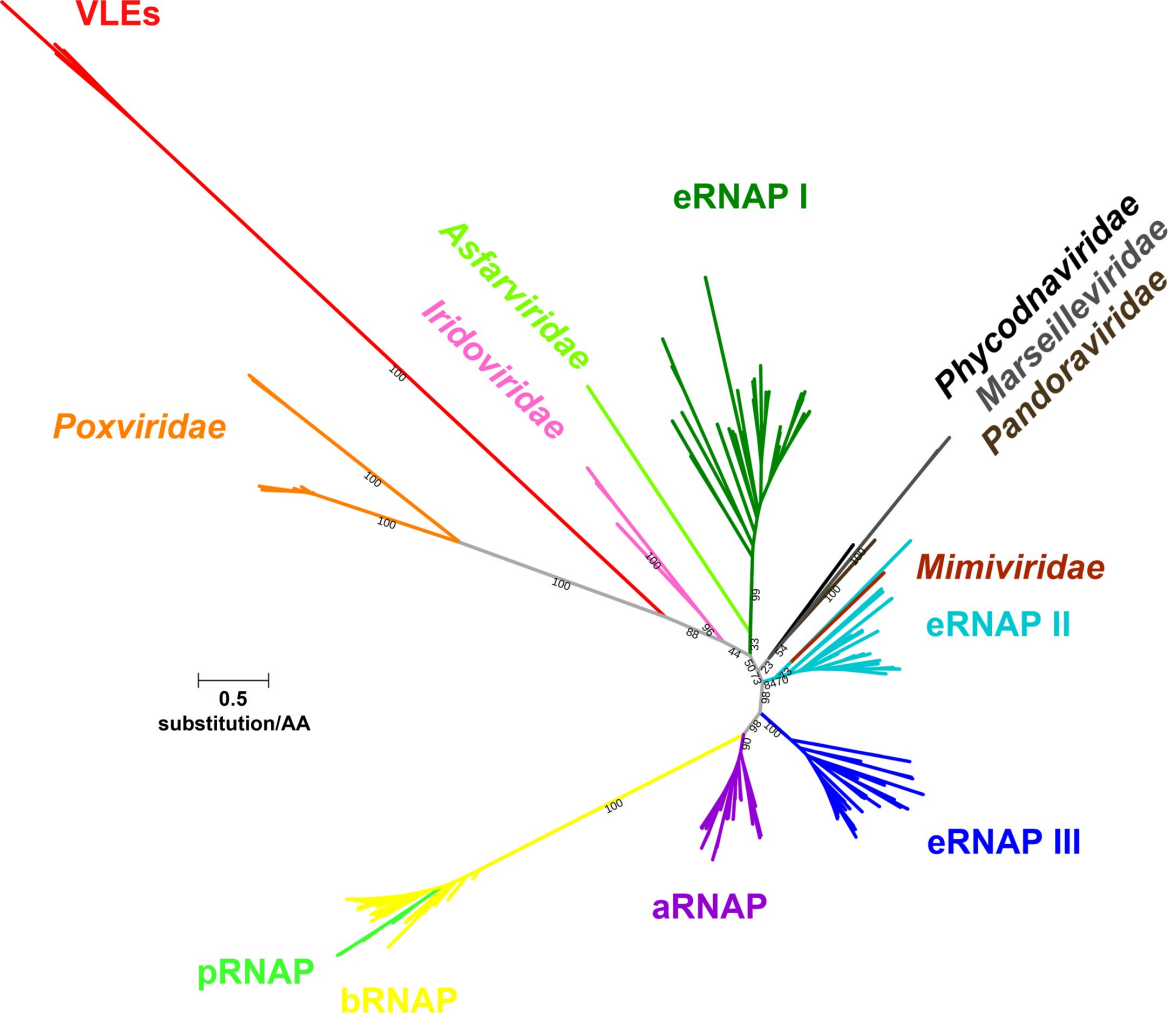
Phylogenetic analysis of yeast VLE RNA polymerases with canonical RNAPs. A phylogram of β′ subunit homologs based on amino acid sequence alignment of β′ subunit conserved regions of selected canonical RNAPs and those β′ subunit conserved regions present in *ORF6* and *ORF7* genes of the yeast VLEs. The maximum likelihood unconstrained tree is displayed as an unrooted phylogram where the length of the branches is proportional to the calculated evolutionary distance of individual sequences. Leaves defining the different classes of RNAPs are labeled. Used abbreviations for RNAP groups: aRNAP, archaeal RNAP; bRNAP, bacterial RNAP; eRNAP, eukaryotic RNAP; pRNAP, plastid RNAP. Length scale of branches of an average value of 0.5 substitution per amino acid residue is shown as a line near the tree. Selected branch support values calculated from 1 000 ultrafast bootstrap replicates optimized using nearest neighbour interchange (NNI) to reduce overestimating support are indicated in black. For details concerning phylogenetic analysis see Materials and methods.

Fig 7 shows the unconstrained phylogenetic tree for conserved β′ subunit regions. The tree surprisingly suggests monophyly of VLE RNAPs with viral RNAPs of the *Poxviridae* family, with viral RNAPs of the *Iridoviridae* family also belonging to the same clade. It is believed that ancestral β′ subunit orthologs of all nucleo-cytoplasmic large DNA viruses (NCLDVs) were monophyletic, but the ancestral gene was displaced in Asfarvirus and Mimivirus for eukaryotic RNAP I and RNAP II gene, respectively [47]. In our analysis, Asfarvirus RNAP clustered with eukaryotic RNAPs I, and Mimivirus RNAP clustered with eukaryotic RNAPs II (Fig 7), which is consistent with that view. Interestingly, a single NCLDV clade (with the exception of *Asfarviridae* and *Mimiviridae*) was not recovered in our unconstrained tree. Further, we tested alternative evolutionary hypotheses for VLE RNAPs *via* constrained tree topology approach where we enforced monophyly of VLE RNAPs with other viral and cellular RNAPs, and we compared the likelihood of the original tree with the likelihood of the constrained trees. From these analyses, it was apparent that the likelihood of the unconstrained tree suggesting monophyly of the yeast VLE RNAP with poxviral RNAP was the best, although monophyly of VLE RNAP with RNAP of other NCLDVs could not be rejected by statistical tests (S7 Fig). Importantly, monophyly of VLE RNAP with eukaryotic RNAP I and RNAP II was rejected at a statistically significant level based on Expected Likelihood Weight test (S7 Fig).

Taken together, our phylogenetic analysis surprisingly points to a viral origin of VLE RNAPs, possibly close to poxviruses, rather than to a cellular origin, contradicting previous hypotheses about the origin of these enzymes [1, 23, 45].

### VLE promoters have a viral origin

Although the UCS (5′-ATNTGA-3′) essential for transcription of VLEs was identified a long time ago, no similarities with known promoters that would indicate its origin were reported. We extended the UCSs preceding all pGKL-encoded ORFs both upstream and downstream by ~10 bp, and we created a consensus motif. This consensus was then used to search for similar elements that were associated with transcription by multisubunit RNAPs. We particularly focused on promoters of viral RNAPs because our phylogenetic analysis of VLE RNAPs had suggested a viral origin.

**Fig 8:**
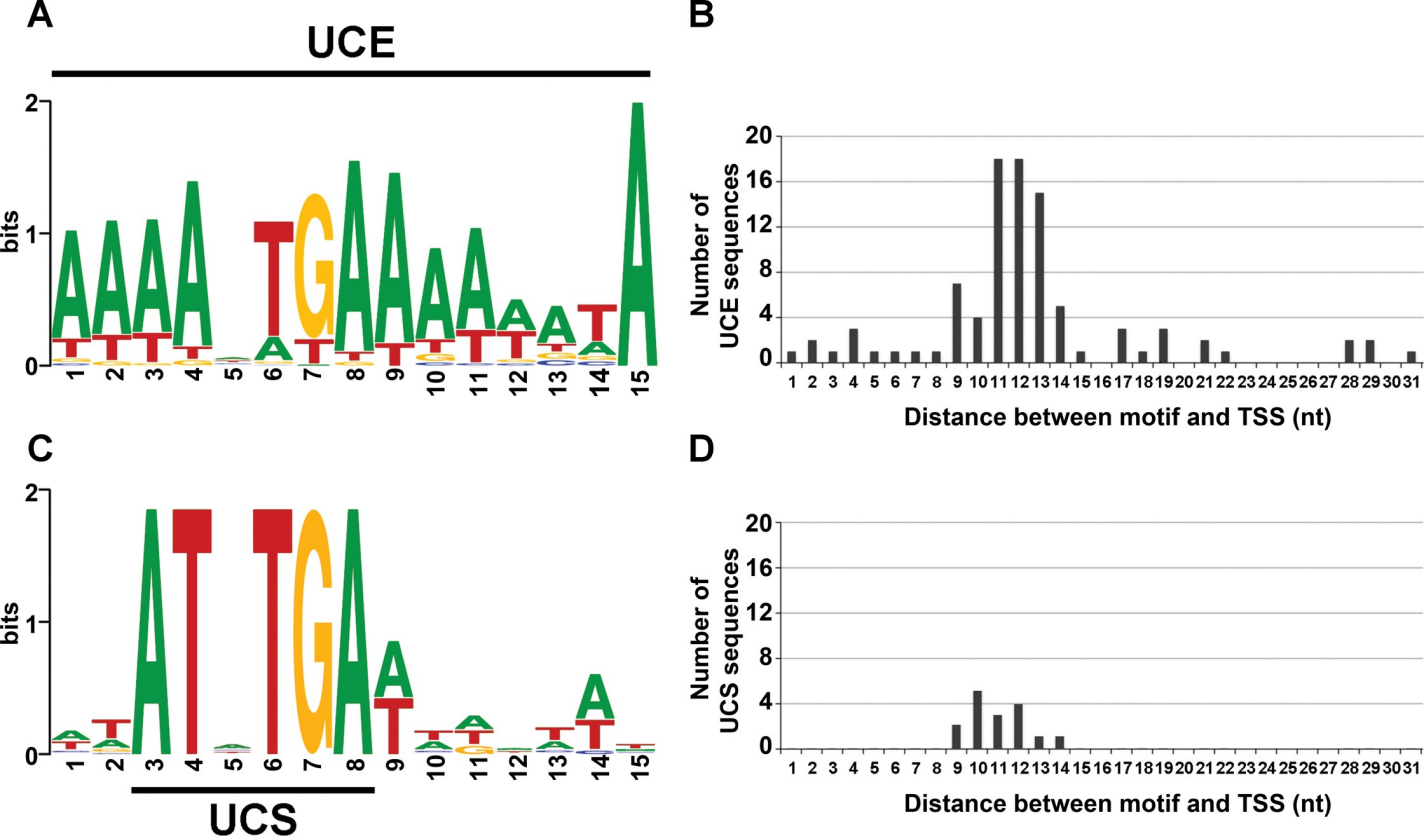
Upstream promoter elements of yeast VLEs show high sequence similarity to promoters of poxviral early genes. (A) Vaccinia virus early promoter consensus motif termed upstream control element (UCE) calculated from 84 sequences identified from genome-wide RNA-sequencing experiments in ref. [48]. (B) Graph showing the number of UCE sequences as a function of their distances to the transcription start sites (TSS) of 84 ORFs as annotated in ref. [48]. (C) Extended promoter consensus motif of pGKL elements preceding 15 ORFs. This motif contains the upstream conserved sequence (UCS) which is universal among yeast VLEs. (D) Graph showing the number of extended UCS sequences as a function of their distances to the TSS of 15 pGKL-encoded ORFs as determined in 5′ RACE-PCR experiments. For promoters with a putative initiator region the first adenosine residue in the region was considered to be the TSS. For more information concerning promoter characterization see Materials and methods.

Notably, we detected great sequence similarity between the extended UCS (Fig 8C) and the upstream control element (UCE), which is a promoter element of poxviral early genes (Fig 8A). The UCE motif is a 15-nt long AT-rich element with any nucleotide at the 5^th^ position followed by TGA [48]. This perfectly matched the extended UCS motif. The median distances from the 3′ ends of the UCEs to the annotated TSSs of 84 Vaccinia virus ORFs displayed a median distance of 12 nt (Fig 8B) [48]. We annotated the TSSs of all pGKL genes based on our previous 5′ RACE-PCR experiments (Materials and Methods, and S4 Table). The distances from the extended UCSs to the annotated TSSs of 15 pGKL-encoded ORFs (Fig 8D) had a median distance of 11 nt. Moreover, we found an adenosine residue to be the TSS nucleotide in all pGKL-encoded ORFs (S4 Table), similar to the TSSs of the poxviral early genes where purines are the dominant TSS bases [48].

To conclude, it appears that promoters of poxviral early genes and VLE genes are similar both with respect to their sequence and their spacing to the TSSs, implying a common origin.

## Discussion

**Fig 9:**
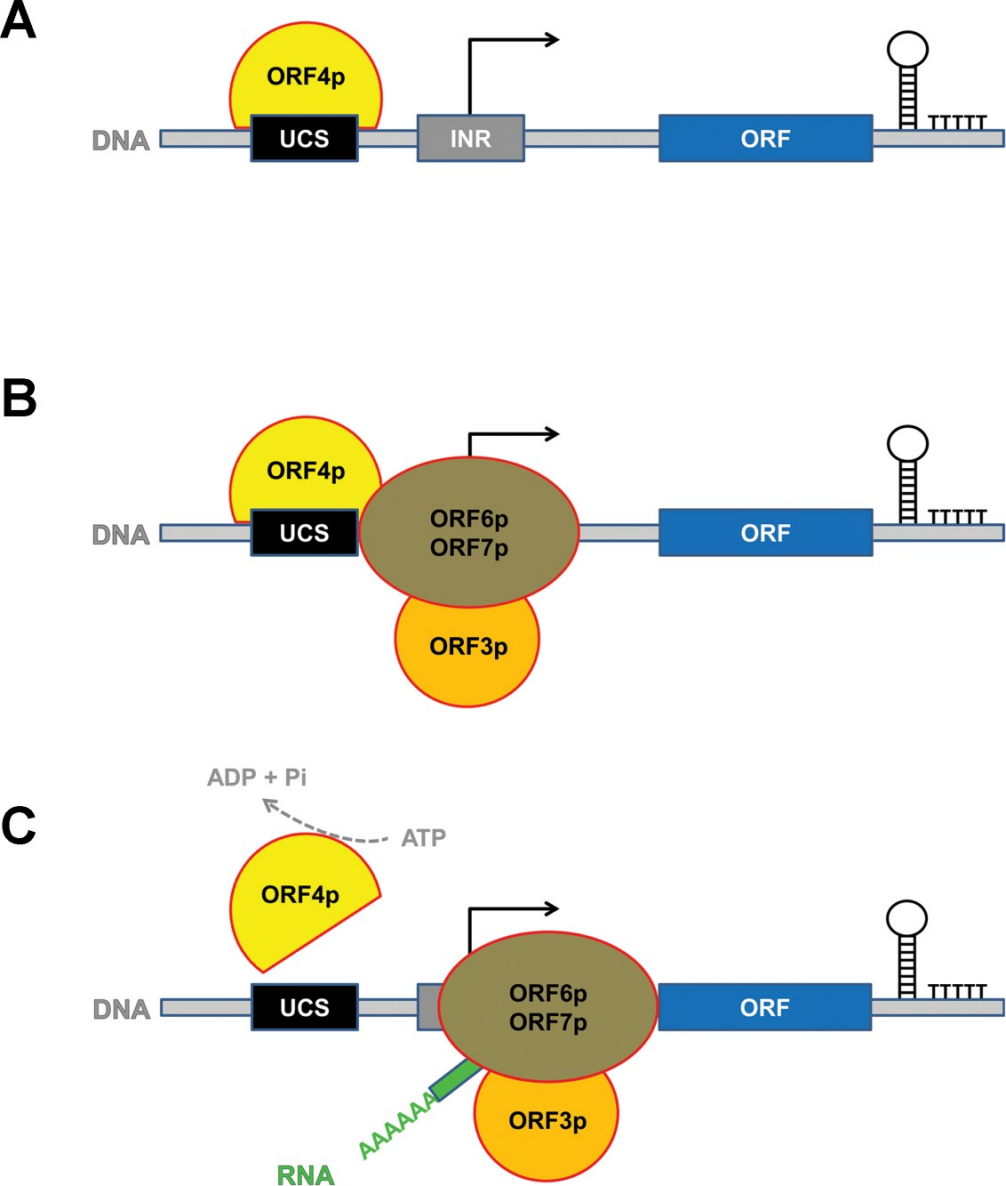
Model of the yeast VLE transcription initiation and termination. (A) Putative helicase K2ORF4p (ORF4p, yellow) binds to the VLE DNA, presumably to the upstream conserved sequence (UCS, black) which is related to the early promoter element of poxviruses. (B) K2ORF4p recruits the RNAP complex (ORF6p/ORF7p, brown) to the transcription initiation site, which usually contains the initiator region (INR, grey) responsible for RNAP slippage and subsequent 5′ mRNA polyadenylation. (C) ATP hydrolysis by K2ORF4p releases it from the transcription preinitiation complex to allow RNAP to escape from the initiation site and produce mRNA (RNA, green) containing a 5′ end poly(A) leader. This RNA can be subsequently 5′ capped by the K2ORF3p viral-like mRNA capping enzyme (ORF3p, orange). Transcription termination most likely proceeds in a factor-independent manner that involves intrinsic terminators consisting of RNA stem loop structure(s) and 3′ terminal U-tract.

In this study we characterized the considerably underexplored transcription machinery of the yeast cytoplasmic linear double-stranded DNA virus-like elements. We used both experimental and bioinformatic approaches, and determined the composition and interactions of the transcription complex and presented a 3D model of its two main subunits. Further, we defined DNA sequences required for initiation and termination. For a model of the key aspects of transcription of the VLEs see Fig 9. Finally, our analyses provided evidence strongly suggesting that poxviruses and the yeast VLEs have a common origin.

### Composition of the VLE transcription machinery

Biochemical characterization of proteins encoded by yeast VLEs was shown to be challenging in the past. Expression of genes located on the pGKL elements seemed to be rather weak [15–17]. Also, it has been shown that expression of the K2ORF3p mRNA capping enzyme in routinely used *E*. *coli* systems was not possible, most likely due to the different codon usage dictated by the high AT content of VLE genes [32]. Recently, it was shown that yeast nuclear expression of VLE genes was impaired because the high AT content of VLE genes led to RNA fragmentation [33]. Accordingly, we failed to express recombinant K2ORF6p and K2ORF7p RNAP subunits in *E*. *coli*, *S*. *cerevisiae* and *K*. *lactis* expression systems.

To overcome this difficulty, we prepared modified and double-modified pGKL elements expressing the transcription components with tags. Using co-immunoprecipitations followed by mass spectrometry and Western blotting we demonstrated that *in vivo* the transcription machinery core complex consisted of the two RNAP subunits (K2ORF6p, K2ORF7p) and the mRNA capping enzyme (K2ORF3p). This interaction was independent of the presence of nucleic acids. Subsequently, we showed that the putative helicase (K2ORF4p) associated less tightly with both the VLE large RNAP subunit and the mRNA capping enzyme. This suggested the following molecular model of the VLE transcription components interactions. First, we propose that K2ORF6p and K2ORF7p interact directly because aa residues of β′a15 and β′a16, which are known to participate in intramolecular (*Bacteria*, *Eukarya*, *Archaea*) or intermolecular (*Archaea*) bonds in multisubunit RNAPs, are present also in K2ORF6p and K2ORF7p as revealed by our *in silico* analysis. Second, we suggest that the mRNA capping enzyme interacts directly with the RNAP complex although it is yet to be determined whether it is with K2ORF6p and/or K2ORF7p. Cellular mRNA capping enzymes are known to interact with the C-terminal domain of RNAP II β′ homolog subunit [49]. However, a homologous C-terminal domain is present neither in Vaccinia virus nor in VLE RNAPs. Nevertheless, in Vaccinia virus, the heterodimeric VTF mRNA capping enzyme interacts directly with the RNAP complex, and it is thought to be present both during transcription initiation and elongation [50, 51]. By analogy, K2ORF3p may utilize a similar mode of interaction with its RNAP. Third, we propose that K2ORF4p binds to the RNAP core complex less tightly than the core subunits do between themselves. Vaccinia virus helicases VETF and NPH-I are known to interact with RNAP through an RNAP-associated protein of 94 kDa (RAP94), and this is specific for RNAP packaged in the virion [52–54]. VETF can interact with RNAP only in the presence of RAP94 [53]. However, there seem to be no RAP94 homologs outside poxviruses, which implies yet another undescribed mechanism of D6/D11 homologs binding to RNAP in other NCLDVs. This might apply also to K2ORF4p of the yeast VLEs. Future studies will have to address the exact mode of K2ORF4p binding to the transcription complex. However, due to relatively weak interaction of the putative helicase with the core of the VLE-specific transcription complex *in vivo*, we assume that it possibly acts as a dissociable transcription factor.

Finally, we found K2ORF4p, K2ORF3p and K2ORF6p to associate with pGKL-specific DNA *in vivo*. We propose that the transcription complex interacts directly with pGKL-specific DNA through RNAP subunits, and that the association of the mRNA capping enzyme with pGKL-specific DNA is indirect. Based on Vaccinia virus early transcription system analogy, we believe that the putative helicase also interacts directly with pGKL-specific DNA with the UCS promoter elements being the K2ORF4p binding sites. However, due to compact genomic organisation of pGKL elements precise mapping of the *in vivo* DNA binding sites of K2ORF4p using ChIP-seq would most likely turn out unsuccessful because resolution of the method would not match close spacing of the UCS elements. Our analysis indeed suggested close spacing of K2ORF4p binding sites *in vivo* because any pGKL region chosen for PCR amplification showed up to be enriched in anti-HA-K2ORF4p ChIP sample. Importantly, it seems that the transcription machinery of the yeast VLEs is remarkably if not entirely self-sufficient, because we did not find any cellular proteins to be specifically associated with the VLE large RNAP subunit using mass spectrometry analysis.

### Transcription initiation

Our previous 5′ RACE-PCR experiments revealed short poly(A) leaders at the 5′ mRNA ends of most pGKL-encoded genes [36]. These 5′ poly(A) leaders were heterogeneous in length among individual transcripts (1–21 adenosines per molecule) and not complementary to the template DNA. Non-template 5′ poly(A) leaders are a characteristic feature of Vaccinia virus intermediate and late gene mRNAs that occur due to slippage of RNAP at the INR of the promoter [37–40]. Moreover, these non-template 5′ poly(A) leaders were also reported for some Vaccinia virus early genes [48, 55, 56]. Thus, it seems that it is a general feature of Vaccinia virus RNAP to slip on consecutive T residues in the INR element notwithstanding the promoter class [48].

It has been shown that 5′ untranslated regions composed of 5′ poly(A) leader sequences prior to start codon have a regulatory role in translation initiation [57, 58]. Using bioinformatics, we detected a putative INR element in promoters of pGKL genes whose transcripts were 5′ polyadenylated. Using 5′ RACE-PCR and mutagenesis of the putative INR, we confirmed that the 5′ poly(A) leader of VLE transcripts was associated with the identified INR element *in vivo*, and it was generated by the same mechanism as shown for Vaccinia virus postreplicative transcripts [39]. In Vaccinia virus, however, mutations in the INR of postreplicative promoters completely abrogate marker gene expression [39, 59]. This was clearly not the case for INR of pGKL promoters because expression of *G418*^*R*^ was used for selection of clones containing recombinant VLEs. To assess possible role of INR alterations on VLE gene expression, we prepared a strain (IFO1267_pRKL1-9) where we introduced TACCC mutations to the TAAAC INR of toxin subunit gene *K1ORF4*, and this strain showed reduced killer toxin production (S8 Fig).

### Transcription termination

We mapped the 3′ mRNA ends of all pGKL-genes using 3′ RACE-PCR experiments. We identified 1–4 putative RNA stem loops close to the 3′ mRNA terminus of each ORF. Although the putative stem loops displayed relatively high values of Gibbs free energy (median of −7.5 kcal/mol), these values were comparable to the genome-wide predicted intrinsic terminators in *Mycoplasma hyopneumoniae* (median of −8.0 kcal/mol), an organism with a similarly high AT content [60]. Further, RNA stem loops of bacterial intrinsic transcription terminators are usually followed by the typical 7–8 nt U-tract that promotes RNAP pausing at weak dA-rU DNA-RNA hybrid [61–63]. Interestingly, we detected T nucleotide enrichment in terminal 8 nt of VLE-specific 3′ cDNA ends that corresponds to putative U-tract (S9 Fig). Importantly, we revealed a direct link between the putative RNA stem loop structure and the transcription termination pattern *in vivo*, suggesting an intrinsic transcription termination model for the yeast VLEs, similar to that in bacteria (reviewed in [64]). Even though we did not analyse the termination efficiency, we assume that transcription reads through at least some of the putative terminator sequences. Otherwise, functional expression of approximately half of the ORFs would not be possible due to the compact genomic organization inherent to pGKL elements. Future experiments will be required to understand this mechanism in more detail.

Even though Vaccinia virus RNAP, presumably related to VLE-specific RNAP, terminates transcription of early genes in a factor-dependent manner it was recently shown that RNA stem loops can influence both efficiency and location of transcription termination *in vitro* [65]. Therefore, proposed substantial reduction of VLE-specific RNAP ancestor might have contributed to adaptation of this enzyme to transcription termination induced by RNA stem loops and possible loss of auxiliary factors required for this process. In Vaccinia virus, the mRNA capping enzyme and NPH-I helicase are the auxiliary factors essential for transcription termination of early genes [26, 31]. Due to similarities of these viral proteins with K2ORF3p and K2ORF4p [25, 28], respectively, the role of VLE mRNA capping enzyme and VLE putative helicase in transcription termination cannot be ruled out because of their interaction with both VLE-specific RNAP and DNA *in vivo*. However, we were not able to locate a specific termination signal (UUUUUNU) of Vaccinia virus early mRNAs that is required for RNAP pausing induced by viral mRNA capping enzyme [65] in VLE mRNAs. This suggests that unlike in poxviral early genes, VLE mRNA capping enzyme possibly does not play a direct role in VLE transcription termination which may instead solely use RNA stem loops for RNAP pausing. Nevertheless, possible roles of K2ORF3p, K2ORF4p, and/or other proteins in transcription termination are yet to be determined.

### *In silico* 3D model of VLE RNAP

Bioinformatic analysis of VLE-specific RNAP proved to be challenging due to its unique reduced architecture and great evolutionary distance from other multisubunit RNAPs. From the 3D model it is evident that VLE RNAP significantly differs from canonical RNAPs in several aspects:

(i) Almost the entire clamp structure element is absent. Only a basal portion of the clamp formed by βa15, βa16 and β′a20 conserved regions is maintained. The clamp is a mobile RNAP element and its closure is important for high stability and processivity of the enzyme. The clamp conformation is regulated by interaction of universally conserved elongation factors NusG and Spt4/5 with the clamp coiled-coil motif [66]–an element likely missing in VLE RNAP. Therefore, it is highly unlikely that VLE transcription machinery could use cellular Spt4/5 to increase processivity.
(ii) The lid and rudder elements are likely missing. The lid acts as a wedge to facilitate dislocation of RNA from the DNA-RNA hybrid molecule, and thereby maintains a constant size of the DNA-RNA hybrid between 7 to 10 base pairs [67]. However, it was shown, that lid-less bacterial and archaeal RNAPs were negatively affected when transcribing from ssDNA but not so much from dsDNA templates [68, 69]. By analogy, RNA displacement from DNA-RNA hybrid molecule should not be affected by absence of the lid in VLE RNAP transcribing double-stranded templates. The lid was also suggested to participate in bacterial intrinsic termination using stem loops [67]. However, bacterial RNAP without the lid was capable of intrinsic termination *in vitro* [69]. Therefore, we hypothesise that this structural feature is not crucial for intrinsic termination by VLE RNAP *in vivo*. The rudder element interacts with the upstream edge of the DNA-RNA hybrid [67]. Experiments using bacterial RNAP with a deleted rudder reported defects in transcription initiation and less stable elongation complexes [70]. This may correlate with the VLE-specific termination of transcription.
(iii) The secondary-channel rim helices are missing. These helices are the binding sites for some transcription factors of multisubunit RNAPs, such as the transcription elongation factor TFIIS [71]. Therefore, it is highly unlikely that VLE transcription machinery could use cellular TFIIS to overcome pause sites and increase proofreading.

### The evolutionary origin of the VLE transcription machinery

A viral origin of the VLE genes encoding the mRNA capping enzyme and the putative helicase was suggested previously [1]. However, the same origin for RNAP genes was not expected because previous hypotheses proposed that those genes originated from ancestral yeast RNAP genes [1, 23] or that they were ancient representatives of multisubunit RNAP diversification due to their simplified architecture [45]. However, no phylogenetic analysis was conducted to support the aforementioned hypotheses. Our results indicate that VLE RNAP has its evolutionary origin close to poxviruses. Poxviral RNAP, such as that of Vaccinia virus, lacks obvious α subunit homologs [72], similarly to VLE RNAP, and is more simplified than eukaryotic RNAPs. Moreover, the constrained tree topology with enforced monophyly of VLE RNAPs with both eukaryotic RNAP I and RNAP II was rejected at a statistically significant level based on Expected Likelihood Weight test. This also correlates with the reported phylogenetic analysis of mRNA capping enzyme guanylyltransferase core regions [1]. Therefore, a reduction of poxviral RNAP instead of yeast RNAP to give rise to the VLE RNAP seems most plausible.

Our promoter analysis suggests that not just the VLE RNAP, but also the VLE promoters are related to nucleo-cytoplasmic large DNA viruses. We noticed sequence similarity between the UCE motif of Vaccinia virus early genes and the extended UCS motif of pGKL elements, as well as their similar location prior to the TSSs. Invariant G residue and several A residues in the minor groove were proposed to be the UCE nucleotides contacted by Vaccinia virus VETF helicase [27]. Presence of the invariant G residue and AT residues at other positions of the extended UCS motif suggests that K2ORF4p might contact UCS of the pGKL elements in a VETF-like fashion and act as an ATP-dependent transcription initiation factor. A sequence motif similar to UCE has been also identified in promoters of early genes of NCLDVs of *Mimiviridae* family, although its location prior to the TSSs seems to differ [73].

A recent evolutionary hypothesis suggests that both yeast VLEs and nucleo-cytoplasmic large DNA viruses branched from a Polintovirus—eukaryotic dsDNA virus that had acquired transcription machinery genes required for escape from nucleus to cytoplasm [74]. Yeast VLEs and NCLDVs then may have evolved separately [74]. It is difficult to dispute this hypothesis, also considering that transcription machinery genes similar to those encoded by the yeast VLEs and NCLDVs have not been reported yet in the so far characterized Polintoviruses. Based on all our results, we propose that yeast VLEs might have evolved directly from a proto-poxvirus rather than from a common ancestor of VLEs and NCLDVs.

In conclusion, the transcription apparatus of the yeast VLEs has most likely an origin close to poxviruses and uses transcription initiation mechanisms similar to those used by poxviral genes. Unlike poxviruses, however, VLEs are beneficial for the cell, and this exemplifies the ability of the cell to domesticate potentially pathogenic elements.

## Materials and methods

### Strains, plasmids and growth conditions

All of the strains used in this study are listed in Table 2. *Escherichia coli* cells were grown at 37°C in 2xTY medium which was supplemented with kanamycin (50 μg/ml) or ampicilin (100 μg/ml) for selection of transformants. Transformations of *E*. *coli* cells were performed by electroporation using Gene Pulser Xcell (Bio-Rad). *K*. *lactis* cells were grown at 28°C in YPD medium which was supplemented with G418 (250 μg/ml) and/or hygromycin B (200 μg/ml) for selection of transformants. Transformations of *K*. *lactis* cells were performed using the one-step LiCl method [75] and followed by five-hour incubation in non-selective conditions immediately after transformation. For detailed descriptions of plasmids and elements used in this study see S1 Table. Constructed pGKL elements were verified by PCR and subsequent sequencing of amplified products.

**Table 2:** List of strains used in this study.

Strain	Genotype	Reference / Source
***E*. *coli* strains**		
XL-1 Blue	*endA1 gyrA96*(nal^R^) *thi-1 recA1 relA1 supE44* *lac* [F' Tn*10*(tet^R^) *proAB lacI*^*q*^*Δ(lacZ)M15*] *hsdR17*(r_K_^-^ m_K_^+^)	Stratagene
***S*. *cerevisiae strains***		
S6/1	MATα	[76]
***K*. *lactis* strains**		
IFO1267	wild-type MATa pGKL1^+^ pGKL2^+^	Institute for Fermentation, Osaka
IFO1267_pRKL1-1	pRKL1-1^+^ (K1UCR2-G418^R^, orf2Δ) pGKL2^+^	This study
IFO1267_pRKL1-2	pRKL1-2^+^ (K1UCR2*-G418^R^, orf2Δ) pGKL2^+^	This study
IFO1267_pRKL1-3	pRKL1-3^+^ (K1UCR2**-G418^R^, orf2Δ) pGKL2^+^	This study
IFO1267_pRKL1-4/2-4	pRKL1-4^+^ (ORF4-HA, HygB^R^) pRKL2-4^+^(yEGFP3-ORF6, G418^R^)	This study
IFO1267_pRKL1-5	pRKL1-5^+^ (G418^R^-K2ORF5 3′ UTR, orf2Δ) pGKL2^+^	This study
IFO1267_pRKL1-6	pRKL1-6^+^ (G418^R^-K2ORF5 3′ UTR°, orf2Δ) pGKL2^+^	This study
IFO1267_pRKL1-7	pRKL1-7^+^ (G418^R^-K2ORF5 3′ UTR°°, orf2Δ) pGKL2^+^	This study
IFO1267_pRKL1-9	pRKL1-9^+^ (K1UCR4**-ORF4, HygB^R^) pGKL2^+^	This study
IFO1267_pRKL2-3	pGKL1^+^ pRKL2-3^+^ (FLAG-ORF6, G418^R^)	This study
IFO1267_pRKL2-4	pGKL1^+^ pRKL2-4^+^ (yEGFP3-ORF6, G418^R^)	This study
IFO1267_pRKL2-5	pGKL1^+^ pRKL2-5^+^ (yEGFP3-ORF6, ORF7-FLAG, G418^R^, HygB^R^)	This study
IFO1267_pRKL2-6	pGKL1^+^ pRKL2-6^+^ (yEGFP3-ORF6, ORF3-HA, G418^R^, HygB^R^)	This study
IFO1267_pRKL2-7	pGKL1^+^ pRKL2-7^+^ (yEGFP3-ORF6, HA-ORF4, G418^R^, HygB^R^)	This study
IFO1267_pRKL2-8	pGKL1^+^ pRKL2-8^+^ (yEGFP3-ORF4, G418^R^)	This study
IFO1267_pRKL2-9	pGKL1^+^ pRKL2-9^+^ (yEGFP3-ORF4, HA-ORF6, G418^R^, HygB^R^)	This study
IFO1267_pRKL2-10	pGKL1^+^ pRKL2-10^+^ (yEGFP3-ORF4, ORF3-HA, G418^R^, HygB^R^)	This study
IFO1267_pRKL2-11	pRKL2-11^+^ (ORF3-yEGFP3, G418^R^)	This study
IFO1267_pRKL2-12	pGKL1^+^ pRKL2-12^+^ (HA-ORF6, HygB^R^)	This study
IFO1267_pRKL2-13	pGKL1^+^ pRKL2-13^+^ (HA-ORF4, HygB^R^)	This study
IFO1267_pRKL2-14	pGKL1^+^ pRKL2-14^+^ (ORF3-HA, HygB^R^)	This study
IFO1267_pRKL2-15	pGKL1^+^ pRKL2-15^+^ (ORF7-FLAG, HygB^R^)	This study

Detailed description of all plasmids/elements used in this study is listed in S1 Table. UCR is the sequence extending from the AUG initiation codon up to and including the UCS of the selected ORF.

*UCR sequence bearing one point mutation in putative initiator region (INR).

**UCR sequence bearing two point mutations in putative initiator region (INR).

°3′ UTR of K2ORF5 gene bearing mutations in putative Stem loop 2.

°°3′ UTR of K2ORF5 gene bearing rescue mutations in putative Stem loop 2.

The nucleotide sequences of the primers used for construction, verification and sequencing of recombinant pGKL elements, and RACE-PCR amplification are listed in S2 Table. All polymerase chain reactions (PCRs) were performed using Taq DNA polymerase (Roche). PCRs for construction of recombinant pGKL elements were performed using mixture of Taq DNA polymerase (Roche) and Pwo DNA polymerase (Roche) in a 99:1 volume ratio, respectively.

### Modification of pGKL elements using homology recombination *in vivo*

*K*. *lactis* IFO1267 strain was transformed with PCR-generated fragment consisting of 5′ and 3′ ends homologous to the part of the pGKL element to be modified and non-homologous part that introduced purification and/or detection tag (yEGFP3, HA-tag, FLAG-tag) into VLE-specific ORF together with a gene encoding resistance marker (G418 or hygromycin B) whose expression is driven by pGKL1-derived upstream control region (UCR, the sequence extending from the AUG initiation codon up to and including the UCS of the selected ORF). This type of construct was prepared by PCR or fusion PCR methodology (for details see S3 Table).

After PCR amplification and gel electrophoresis, corresponding fragments were purified using FavorPrep GEL/PCR Purification Kit (Favorgen) and used for transformation. After transformation, yeast cells were plated onto selective media and analyzed using agarose electrophoresis for the presence of the modified pGKL element. Usually, it was possible to detect both, modified and wild-type target element directly after transformation. Colonies containing both (modified and wild-type variants of the target pGKL element) were selected and cultivated under selective conditions for approximately 60 generations and analyzed again using agarose electrophoresis. For subsequent analysis and preparation of double-modified pGKL elements, colonies containing only modified variant of the target pGKL element were used. Absence of unmodified variant of the respective pGKL element was also verified by PCR and subsequent electrophoresis.

### Isolation of pGKL elements and electrophoresis

Modified protocol based on [77] was used for analysis of the pGKL VLEs. Briefly, cells were grown on YPD plates supplemented with G418 and/or hygromycin B for three days, transferred into a microplate well and dried for 2 hours at 45°C. After complete drying the cells were resuspended in 40 μl of freshly prepared TESP buffer (20 mM Tris-HCl, pH 8, 50 mM EDTA, 2% SDS, 0.5 mg/ml pronase E) and dried overnight at 37°C. The next day the sample was completely resuspended in 40 μl of 1x DNA loading buffer (Fermentas). 15 μl of the sample was analyzed by electrophoresis (0.5% agarose, voltage 1 V/cm) for at least 20 hours. Subsequently, the gel was incubated in a solution containing ethidium bromide (0.5 μg/ml) and RNase A (50 μg/ml) for at least 3 hours, and then briefly incubated in distilled water.

### Co-immunoprecipitation and mass spectrometry

For the co-immunoprecipitation experiments, 100 ml of the yeast cells from the late exponential growth phase (OD_600_ = 4–8) were used. The cells were harvested with centrifugation, washed with distilled water and lysed in 2–3 ml of ice-cold GFP-wash buffer (10 mM Tris-HCl, pH 7.5, 150 mM NaCl, 0.5 mM EDTA) supplemented with 1 mM PMSF, cOmplete Mini protease inhibitors (Roche) and 0.45 mm glass beads using Mixer Mill MM 301 (Retsch) at a frequency of 30/s for 5 min. The glass beads and cell debris were pelleted with centrifugation at 8 000 g for 5 min at 4°C. The lysates were clarified with centrifugation at 20 000 g for 20 min at 4°C. Co-immunoprecipitations were performed using 25 μl of GFP-Trap_A (Chromotek) beads with gentle mixing overnight at 4°C. Mock immunoprecipitations were performed using 25 μl of empty agarose beads (Chromotek) with gentle mixing overnight at 4°C. Bound proteins were washed three times in 1 ml of ice-cold GFP-wash buffer with gentle mixing for 5 min at 4°C. The immunoprecipitated complexes were dissolved in 60 μl of 2X sample loading buffer (0.1 M Tris-HCl, pH 6.8, 20% glycerol, 2% β-mercaptoethanol, 4% SDS and 0.04% bromophenol blue), incubated for 5 min at 95°C and subjected to SDS-PAGE. The gel was stained with Coomassie Brilliant Blue G-250 or silver. Gel lanes or bands of interest were excised from the gel, digested with trypsin [78], and analysed by mass spectrometry. Identity of all detected proteins was also confirmed by MS/MS analysis. To test, whether certain co-immunoprecipitation is dependent on nucleic acids, the bound washed proteins were treated with 25U of Benzonase Nuclease (Novagen) in GFP-wash buffer for 30 min at 33°C and washed three times again, prior to SDS-PAGE analysis. Fraction of the bound washed proteins was taken and nucleic acids were eluted in 30 μl of TE buffer (10 mM Tris-HCl, pH 8.0, 1 mM EDTA), incubated for 5 min at 95°C, and 1 μl of the nucleic acids was used for detection of DNA using PCR amplification for 22–25 cycles and subsequent electrophoresis.

### Chromatin immunoprecipitation

For the chromatin immunoprecipitation experiments, 50 ml of the yeast cells from the late exponential growth phase (OD_600_ = 4–8) were used. The cells were cross-linked with formaldehyde (1% final concentration) added to the growing culture for 40 min at 28°C. The cells were harvested with centrifugation, washed with distilled water and lysed in 2 ml of ice-cold non-denaturing lysis buffer (50 mM Tris-HCl, pH 7.5, 300 mM NaCl, 5 mM EDTA, 1% Triton X-100, 0.02% sodium azide) supplemented with 1 mM PMSF, cOmplete Mini protease inhibitors (Roche) and 0.45 mm glass beads using Mixer Mill MM 301 (Retsch) at a frequency of 30/s for 5 min. The lysate was sonicated (Qsonica Ultrasonic Processor Q700, 50% amplitude) sixty times with 10 sec pulses. The glass beads and cell debris were pelleted with centrifugation at 8 000 g for 5 min at 4°C. The lysates were clarified with centrifugation at 20 000 g for 20 min at 4°C and 50 μl aliquot of the clarified lysates representing chromatin before immunoprecipitation (Input) was taken. The DNA fragments were immunoprecipitated using 30 μl of mouse monoclonal anti-HA HA-7 agarose (Sigma Aldrich) beads or 20 μl of GFP-Trap_A (Chromotek) beads with gentle mixing overnight at 4°C. The beads were then washed once in 1 ml of ice-cold Wash buffer (20 mM Tris-HCl, pH 8.0, 150 mM NaCl, 2 mM EDTA, 1% Triton X-100, 0.1% SDS) supplemented with single stranded salmon sperm DNA (100 μg/ml; Roche), twice in 1 ml of ice-cold Wash buffer, and once in 1 ml of ice-cold Final wash buffer (Wash buffer containing 500 mM NaCl), each time with gentle mixing for 5 min at 4°C. Immunocomplexes were then eluted from the beads in 120 μl of Elution buffer (1% SDS, 100 mM NaHCO_3_) for 30 min at 37°C (anti-HA HA-7 beads) or for 30 min at 65°C (GFP-Trap_A beads). Eluted immunocomplexes and 50 μl of the clarified lysates representing chromatin before immunoprecipitation (Input) were mixed with 400 μl of TBS (50 mM Tris-HCl, pH 7.5, 150 mM NaCl) supplemented with 5 μl of proteinase K (20 mg/ml; Sigma Aldrich), and the cross-linking was reversed by incubation for 5 hr at 65°C. The immunoprecipitated and input DNA was isolated by phenol-chloroform extraction followed by ethanol precipitation supplemented with 1 μl of linear polyacrylamide (25 mg/ml; Sigma Aldrich), and then used for PCR amplification for 25–30 cycles followed by electrophoresis. PCR amplifications were carried out on 1/30 of the chromatin immunoprecipitation (ChIP) and 1/1200 of the chromatin before immunoprecipitation (Input) using primers listed in S2 Table.

### Western blotting

SDS-PAGE gels were electroblotted onto Immun-Blot PVDF Membrane (Bio-Rad). The membranes were blocked in 5% non-fat dry milk (Hero) in a TBS-Tween buffer (50 mM Tris-HCl, pH 7.5, 150 mM NaCl and 0.5% Tween-20) and incubated with a primary antibody overnight at 4°C. After washing in TBS-Tween buffer and blocking with 5% non-fat milk, the membranes were incubated with a goat anti-mouse HRP-conjugated antibody (1:5 000 dilution; Santa Cruz Biotechnology). Finally, after washing in TBS-Tween buffer, the membranes were immersed in a luminol detection solution and the signal was detected using ImageQuant LAS 4000 (GE Healthcare). To confirm the expression of the target protein and the successful immunoprecipitation, a mouse monoclonal anti-FLAG M2 antibody (1:1 000; Sigma Aldrich), mouse monoclonal anti-HA 6E2 antibody (1:1 000; Cell Signaling), and mouse monoclonal anti-GFP B-2 antibody (1:1 000; Santa Cruz Biotechnology) was used.

### RNA isolation, electrophoresis, reverse transcription, 5′ and 3′ RACE-PCR

25 ml of the yeast cells from the exponential growth phase (OD_600_ = 0.5–1) were quickly pelleted and frozen. Total yeast RNA was isolated by the hot acidic phenol procedure followed by ethanol precipitation [79]. Remaining DNA was removed by DNA-*free* Kit (Ambion). The quality of RNA was assessed by electrophoresis and UV spectrophotometry [80].

In the case of 5′ RACE, subsequent reverse transcription was carried out as follows: 1 μg of total yeast RNA and 0.15 μg of random hexamer primers (Invitrogen) were used for cDNA synthesis using 100U of SuperScript III Reverse Transcriptase (Invitrogen). The cDNA was purified using High Pure PCR Product Purification Kit (Roche) and used for cDNA tailing using 800U of rTdT (Fermentas) and 0.5 mM dGTP (Roche) in 50 μl reaction for 30 min at 37°C with subsequent heat inactivation of rTdT for 10 min at 70°C. For PCR amplification of cDNA ends, 2.5 μl of the reaction mixture was used with olig2(dC)anchor primer and appropriate gene-specific primer for 35 cycles.

In the case of 3′ RACE, 1 μg of total yeast RNA was polycytidinylated using Poly(A) Tailing Kit (Applied Biosystems) and 2 mM CTP (Thermo Scientific) for 90 min at 37°C. Following reverse transcription was performed using 100U of SuperScript III Reverse Transcriptase (Invitrogen) and 1 μg of oligo(dG)anch2 primer. The cDNA was purified using High Pure PCR Product Purification Kit (Roche) and 2.5 μl of the purified cDNA was used for PCR amplification of cDNA ends with anch2 primer and appropriate gene-specific primer for 35 cycles.

In both RACE experiments, after PCR amplification and electrophoresis, obtained fragments were verified using restriction digestion and fragments exhibiting correct digestion pattern were gel-purified using FavorPrep GEL/PCR Purification Kit (Favorgen) and cloned to the pCR4-TOPO vector (Invitrogen). Vectors were transformed into *E*. *coli* XL-1 Blue cells (Stratagene), isolated using GenBond Plasmid FlexSpin Kit (Renogen Biolab) and sequenced using universal T7 promoter primer or T3 primer.

### Structure modelling

Modelling of the tertiary structure of the non-canonical RNA polymerase of the pGKL elements was carried out on the Robetta server [81]. Selection of the optimal template for comparative modelling by Robetta is automatic and five structural models for each sequence are given as the output. All predicted models were compared according to their tertiary structure with a database of known structures using DaliLite v. 3 [82]. Models that gave the best Z-scores to the known structures of canonical RNA polymerases were used for further analyses. In order to prevent partial misalignment of the sequences with the templates used for modelling, sequences of the K2ORF6p (P05472.1) and K2ORF7p (P05473.1) had to be split into parts that were submitted separately for the modelling.

Amino acid residues 1–693 of K2ORF6p were modelled by β subunit homolog of *Schizosaccharomyces pombe* RNAP II (PDB ID: 3H0G.B), residues 754–882 were modelled by β′ subunit of *Thermus thermophilus* RNAP (PDB ID: 2A6H.D), and residues 894–974 were modelled by β′ subunit homolog of *S*. *pombe* RNAP II (PDB ID: 3H0G.A). Amino acid residues 1–52 of K2ORF7p were modelled by β′ subunit homolog of *S*. *pombe* RNAP II (PDB ID: 3H0G.A), and residues 103–132 were modelled by β′ subunit homolog of *Saccharomyces cerevisiae* RNAP II (PDB ID: 1TWF.A).

Models of each part of K2ORF6p and K2ORF7p with the best Z-scores from previous comparison using DaliLite v. 3 were individually superimposed to *S*. *cerevisiae* RNA polymerase II elongation complex (PDB ID: 2NVQ) using DaliLite v. 3 in pairwise option and visualized together using PyMOL 1.3 software (The PyMOL Molecular Graphics System, Version 1.3 Schrödinger, LLC.). The template modeling score (TM-score) value [83], which measures the structural similarity, was calculated for each model:structure superimposition using TM-score (http://zhanglab.ccmb.med.umich.edu/TM-score/). Methionine 9 residue of the K2ORF6p sequence was considered to be the methionine 1 residue because it is thought that the second ATG within the ORF is the true start codon [6]. Therefore, numbering of K2ORF6p residues goes accordingly in this work.

### Phylogenetic analysis

Phylogenetic analysis was performed using β′ subunit conserved sequence regions of multisubunit RNAPs that are present in all canonical RNAPs together with corresponding regions that were found in yeast VLE RNAPs. A single amino acid (aa) sequence was created by joining the aa sequences of K2ORF6p and K2ORF7p for each of the following organisms: *Kluyveromyces lactis* (P05472.1 and P05473.1), *Saccharomyces kluyveri* (CAA38625.1 and CAA38626.1), *Pichia acaciae* (CAJ57280.1 and CAJ57281.1) and *Pichia etchellsii* (CAC08226.1 and CAC08227.1). The joined sequences of RNAP subunits were aligned using default settings of Clustal Omega [84]. β subunit parts together with amino acid residues that did not correspond to previously [23, 24] identified β′ subunit conserved regions were removed. Alignments of VLE RNAP β′ subunit conserved regions were combined manually with previously published alignments [46] of β′ subunit conserved regions of eukaryotic (120 sequences), archaeal (33 sequences), bacterial (40 sequences), plastid (4 sequences) and viral (34 sequences) RNAPs. Sequences of *Lausannevirus* (YP_004346983.1 and YP_004346982.1), *Marseillevirus marseillevirus* (YP_003406803.1 and YP_003406800.1), *Pandoravirus dulcis* (YP_008318947.1), and *Pandoravirus salinus* (YP_008436862.1) RNAP β′ subunits were aligned using default settings of Clustal Omega, and their β′ conserved regions were then manually added to the final alignment. PhyML 3.0 with Smart Model Selection [85] was used for initial maximum likelihood phylogenetic tree construction and selection of a substitution matrix and a model for rates across sites. For detailed phylogenetic analysis, unconstrained and alternative (constrained) topology maximum likelihood trees were constructed using IQ-TREE 1.6.1 [86] with the substitution model (LG+I+G4) found to be the best in the first-round analysis. Tree topologies were compared with IQ-TREE 1.6.1 using the Expected Likelihood Weight (ELW) test, and the approximately unbiased (AU) test. iTOL 3 [87] was used to view and analyze the phylogenetic trees.

### Promoter characterization

The known UCSs preceding all 15 pGKL-encoded genes [8] were used to construct manual alignments including sequences adjacent to the UCS at both upstream and downstream ends. WebLogo 2.8.2 (http://weblogo.berkeley.edu/logo.cgi) was used to create a consensus motif. The literature was searched for elements with similar motives known to be associated with promoter activity of multisubunit RNAPs.

Transcription start sites (TSSs) were annotated according to our previous 5′ RACE-PCR experiments covering all pGKL genes [36]. Briefly, sequencing of individual cDNA clones of *K2ORF2*, *K2ORF3* and *K2ORF8* transcripts displayed a single peak TSS pattern, where more than 60% clones had the same initiator nucleotide and other initiator nucleotides were not represented in more than 25% of the clones. Sequencing of cDNA clones corresponding to transcripts of other pGKL-encoded ORFs displayed a multiple peaks TSS pattern, due to presence of a non-templated 5′ poly(A) leader of a heterogeneous length. For those ORFs the first adenosine residue of the clones that could be aligned to the promoter template sequence was considered to be the initiator nucleotide.

### Assay of killer toxin activity

Filter sterilized culture medium was tested for the presence of the killer toxin activity by the agar well diffusion assay using *S*. *cerevisiae* S6/1 as a sensitive strain. Approximately 2x10^5^ of sensitive yeast cells were plated onto YPD plates (1% yeast extract, 2% peptone, 2% glucose, 2% agar) for testing of pGKL1 killer toxin activity. Wells were made with an 8 mm diameter cork borer and 100 μl of filter sterilized culture medium or 100 μl of serial dilution of the filter sterilized culture medium was pipetted into well.

## Supporting information

S1 Fig Electrophoretograms of isolated pGKL elements from yeast strains used in this study. For each electrophoretogram DNA mass markers are indicated on the left, and native or recombinant pGKL elements are indicated on the right side, respectively. Recombinant pGKL elements are marked in red. M1, DNA molecular mass marker (Lambda DNA/Eco130I (StyI) Marker, Fermentas). M2, DNA molecular mass marker (GeneRuler 1 kb DNA Ladder, Thermo Scientific). IFO1267—*K*. *lactis* strain with wild-type pGKL elements.
(TIF)

S2 Fig Identification of proteins associated with the capping enzyme encoded by the yeast VLEs. (A) IFO1267_pRKL2-4 (containing yEGFP3-K2ORF6p) and IFO1267_pRKL2-11 (K2ORF3p-yEGFP3) cells were grown to late exponential phase. The cells were lysed, yEGFP3-K2ORF6p and K2ORF3p-yEGFP3 were affinity-purified using GFP-Trap agarose beads. Bound proteins were digested with trypsin, and then analyzed by mass spectrometry. Also, an aliquot of the beads was taken and bound proteins were eluted and resolved by SDS-PAGE, and the gel was silver-stained. Proteins identified by mass spectrometry are indicated with arrows on the right side. Proteins identified by mass spectrometry in previous experiments described in Table 1 and Fig 1 are indicated with dashed arrows on the right side. Bands corresponding to small RNA polymerase subunit were not clearly visible, presumably due to their smaller mass and weaker staining. M, protein molecular mass marker (PageRuler Prestained Protein Ladder, Fermentas); the respective molecular mass values are indicated on the left side. (B) K2ORF3p-associated proteins identified by mass spectrometry. The proteins identified, their molecular weight (MW), unique coverage, and the number of peptide types from IFO1267_pRKL2-11 strain is listed.
(TIF)

S3 Fig Physical association of the putative helicase and capping enzyme with the large RNAP subunit of the yeast VLEs is not dependent on nucleic acids. (A) yEGFP3-K2ORF6p was purified with GFP-Trap agarose beads from lysates of the IFO1267_pRKL2-6 strain cells. After washing the bound immunoprecipitated proteins (α-GFP IP), the beads were split into two parts which were mock-treated (Benz-) and treated (Benz+) with Benzonase Nuclease to digest DNA and RNA. Then, the beads were extensively washed again and the bound proteins were eluted and analysed by Western blotting using anti-GFP (α-GFP) and anti-HA (α-HA) antibodies. Also, an aliquot of the beads was taken and bound nucleic acids were eluted and analyzed for presence of pRKL2-6 VLE DNA by PCR amplification for 25 cycles and visualized by electrophoresis. M, DNA molecular mass marker (GeneRuler 100 bp Plus DNA Ladder, Fermentas); respective molecular mass values of two DNA fragments (500 bp and 1 000 bp) are indicated. (B) yEGFP3-K2ORF6p was purified with GFP-Trap agarose beads from lysates of IFO1267_pRKL2-7 strain cells. After washing the bound immunoprecipitated proteins (α-GFP IP), the beads were split into two parts that were mock-treated (Benz-) and treated (Benz+) with Benzonase Nuclease to digest DNA and RNA. Then, the beads were extensively washed again and the bound proteins were eluted and analyzed by Western blotting using anti-GFP (α-GFP) and anti-HA (α-HA) antibodies. Also, an aliquot of the beads was taken and bound nucleic acids were eluted and analyzed for presence of pRKL2-7 VLE DNA by PCR amplification for 22 cycles and electrophoresis. M, DNA molecular mass marker (GeneRuler 100 bp Plus DNA Ladder, Fermentas); respective molecular mass values of two DNA fragments (500 bp and 1 000 bp) are indicated.
(TIF)

S4 Fig 3′ ends of VLE-specific mRNAs are located close to putative RNA stem loop structures. This figure represents results of 3′ RACE-PCR analysis of individual mRNAs corresponding to all genes encoded by the pGKL elements. Total RNA was isolated from wild-type IFO1267 strain, DNase treated and 3′ polycytidinylated. Reverse transcription was carried out using oligo(dG)anch2 primer. Purified cDNA was used for 3′ RACE-PCR using anch2 primer and gene-specific primers listed in S2 Table. After PCR amplification and electrophoresis, the identity of the products was verified using restriction digestion and fragments exhibiting correct digestion pattern were gel-purified, cloned to the pCR4-TOPO vector and sequenced. The upper sequence on the right side corresponds to the template (plasmid) DNA and its position in the pGKL genome is annotated using *K*. *lactis* pGKL1 (X00762.1) and pGKL2 (X07776.1) sequences. Sequences situated below represent individual sequenced cDNA clones (only distal part of 3′ untranslated region is shown). Cytosine residues at the 3′ end of cDNA corresponding to the RNA tail are omitted in this representation for clarity. RNA secondary structures close to 3′ ends of VLE-specific mRNAs were predicted using default settings of RNAstructure Server (http://rna.urmc.rochester.edu/RNAstructureWeb/) [42]. Predicted RNA stem loops are displayed as cDNA nucleotide letters in circles on the left side, and the values of Gibbs free energy (ΔG) in kcal/mol are displayed for each structure. Stem loop distances from gene stop codon are shown as numbers of nucleotides (nt). Final nucleotides of the experimentally determined 3′ ends of cDNA are shown as colored letters enlarged proportionally to their occurrence (in percent) in the sequenced clones when the same final nucleotide was detected in at least two independent clones. Predicted RNA stem loop structures and sequenced cDNA clones are listed as follows: (A) *K1ORF1*, (B) *K1ORF2*, (C) *K1ORF3*, (D) *K1ORF4*, (E) *K2ORF1*, (F) *K2ORF2*, (G) *K2ORF3*, (H) *K2ORF4*, (I) *K2ORF5*, (J) *K2ORF6*, (K) *K2ORF7*, (L) *K2ORF8*, (M) *K2ORF9*, (N) *K2ORF10*, (O) *K2ORF11*.
(PDF)

S5 Fig 3′ ends formation of VLE-specific mRNAs is dependent on 3′ untranslated region and not dependent on promoter and coding sequence of the gene. This figure represents results of 3′ RACE-PCR analysis of individual mRNAs corresponding to the G418 and hygromycin B resistance genes expressed under control of K1UCR2 and K1UCR3, respectively. (A) Schematic representation of recombinant pGKL2 elements where the resistance marker genes are inserted prior to *K2ORF5* gene in the same transcriptional orientation. (B) Total RNA was isolated from IFO1267_pRKL2-3 and IFO1267_pRKL2-12 strains, DNase treated and 3′ polycytidinylated. Reverse transcription was carried out in the presence (+RT) and absence (-RT) of reverse transcriptase using oligo(dG)anch2 primer. Purified cDNA was used for 3′ RACE-PCR using anch2 primer and gene-specific primers listed in S2 Table. After PCR amplification the samples were analyzed in 1.8% agarose gel stained by ethidium bromide. M, DNA molecular mass marker (GeneRuler 100 bp Plus DNA Ladder, Fermentas). The respective values are indicated on the left side. Specific products that were cloned to the pCR4-TOPO vector and used for sequencing are labelled with asterisks. Predicted RNA stem loop structures and sequenced cDNA clones for mRNA 3′ ends of (C) G418 and (D) Hygromycin B resistance genes are depicted as in S4 Fig.
(TIF)

S6 Fig ORF6 proteins of yeast VLEs show sequence similarity to β subunit conserved regions of multisubunit RNAPs. Conserved regions are named according to ref. [46]. In the resulting sequence alignment the identity (black shading) was highlighted where the same amino acid residue occurred in ≥ 50% of the sequences and sequence similarity (gray shading) was highlighted where amino acid residue with similar properties occurred in ≥ 50% of the sequences. The numbers in brackets indicate the number of amino acid residues that were not displayed in this comparison. Sequence identity (in %) of consecutively numbered sequences is depicted in a table below each alignment. Local reliability of sequence alignments was evaluated using the Transitive Consistency Score (TCS) web server [88] and apart from the second half of βa6 conserved region alignment all alignments showed good or average local reliabilities. Following sequences (with their accession numbers) were used for the alignment: *Thermus aquaticus* β (CAB65465.2), *Escherichia coli* β (AAC76961.1) *Methanocaldococcus jannaschii* B′′ (Q58444.1), *Methanocaldococcus jannaschii* B′ (Q60181.1), *Saccharomyces cerevisiae* Rpa135 (CAA95050.1), *Saccharomyces cerevisiae* Rpb2 (NP_014794.1), *Sascharomyces cerevisiae* Rpc128 (CAA99422.1), Vaccinia virus Rpo132 (AAQ93241.1), *Kluyveromyces lactis* ORF6-pGKL2 (P05472.1), *Saccharomyces kluyveri* ORF6-pSKL (CAA38625.1), *Pichia acaciae* ORF6-pPac-1 (CAJ57280.1), *Pichia etchellsii* ORF6-pPE1B (CAC08226.1).
(DOCX)

S7 Fig Results of statistical analysis of constrained trees for the β′ subunit conserved regions of multisubunit RNAPs. The trees are ranked from best to worst based on their likelihood. c-ELW, Expected Likelihood Weight. p-AU, p-value of approximately unbiased (AU) test. logL, log-likelihood. Plus signs denote the 95% confidence sets. Minus signs denote significant exclusion. All tests were performed with 10 000 resamplings using the RELL method.
(XLSX)

S8 Fig Mutations in the initiator region of VLE-specific promoter decrease gene expression. This figure represents results of killer toxin activity assay. IFO1267_pRKL1-9 (TACCC INR) and IFO1267 (control) cells were cultivated in YPD medium at 24°C. Aliquots were taken at 0, 3, 6 and 12 hours, and the culture medium was filter-sterilized, diluted, and assayed for the presence of the killer toxin activity by an agar well diffusion test using a lawn of *S*. *cerevisiae* S6/1 sensitive strain cells grown on YPD plates at 24°C for 2 days. Result from post-cultivation medium taken at 12 hours is shown.
(TIF)

S9 Fig 3′ ends of VLE-specific mRNAs contain putative U-tails. This figure represents results of sequence analysis of all cDNA clones whose sequences were detected in at least two independent clones obtained from the 3′ RACE-PCR analysis of mRNAs corresponding to all genes encoded by the pGKL elements. Those cDNA sequences (58 non-redundant sequences) were manually aligned according to their transcription termination site (TSS), and frequencies of individual nucleotides were calculated for each position within the 50 nt upstream of the TSS using Seqool 3.1 (http://www.biossc.de/seqool/dwnload.html) and plotted. The occurrence of the T residue in the 8 3′ terminal nucleotides of cDNA ends (including TSS) is ≥ 50%.
(DOCX)

S1 Table Plasmids and virus-like elements used in this study. (DOCX)

S2 Table Primers used in this study. (DOCX)

S3 Table Primers, templates, and resulting PCR products used for modifications of pGKL elements. (DOCX)

S4 Table Transcription start site positions. (DOCX)
