# Supplementary figures and images for: Transcription apparatus of the yeast virus-like elements: Architecture, function, and evolutionary origin
Source: PLoS Pathog. 2018 Oct 22;14(10):e1007377. doi: 10.1371/journal.ppat.1007377 (PMC6211774; doi:10.1371/journal.ppat.1007377)

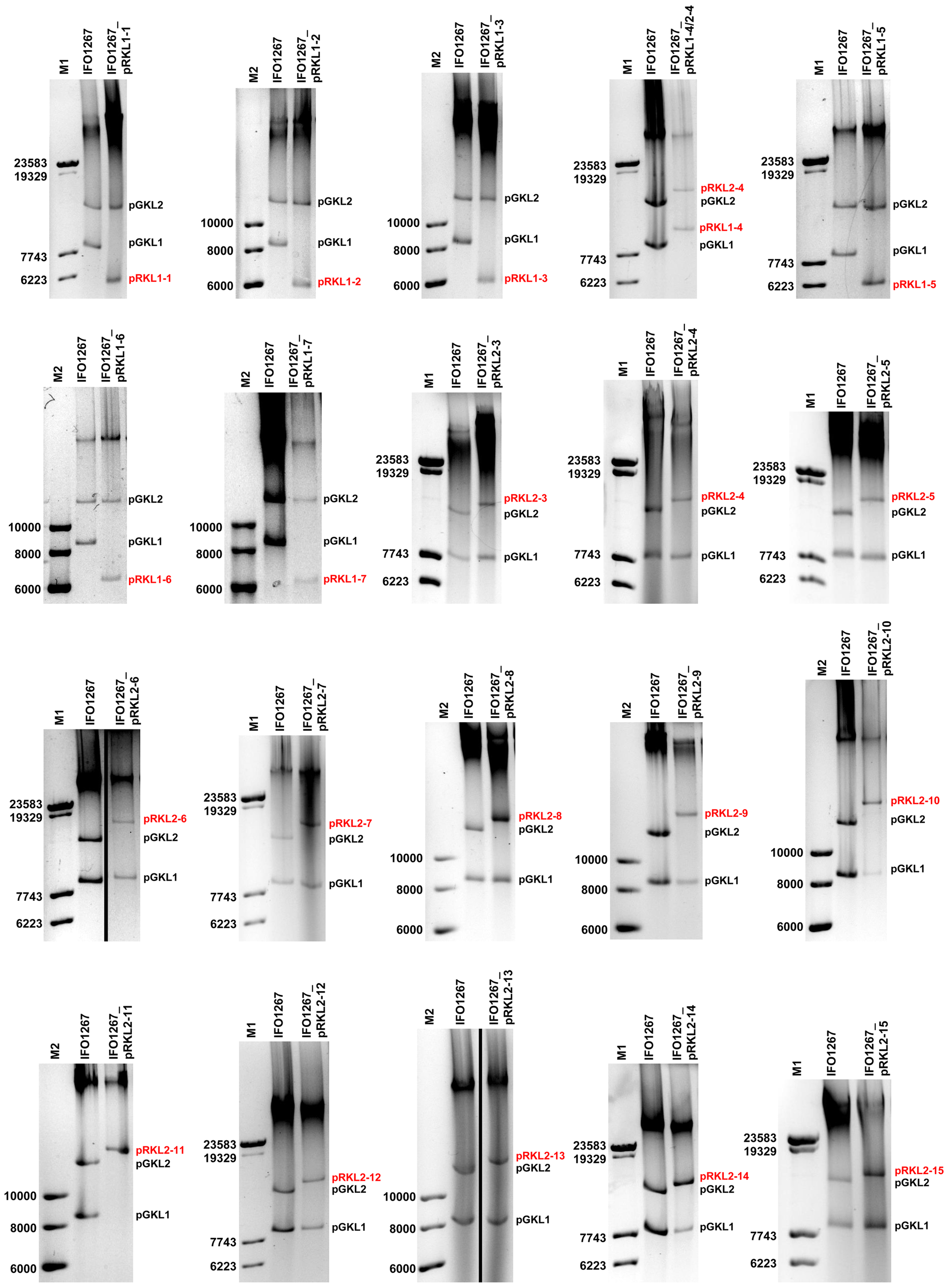

Supplement: S1 Fig — For each electrophoretogram DNA mass markers are indicated on the left, and native or recombinant pGKL elements are indicated on the right side, respectively. Recombinant pGKL elements are marked in red. M1, DNA molecular mass marker (Lambda DNA/Eco130I (StyI) Marker, Fermentas). M2, DNA molecular mass marker (GeneRuler 1 kb DNA Ladder, Thermo Scientific). IFO1267—K. lactis strain with wild-type pGKL elements. (TIF) [file ppat.1007377.s001.tif]

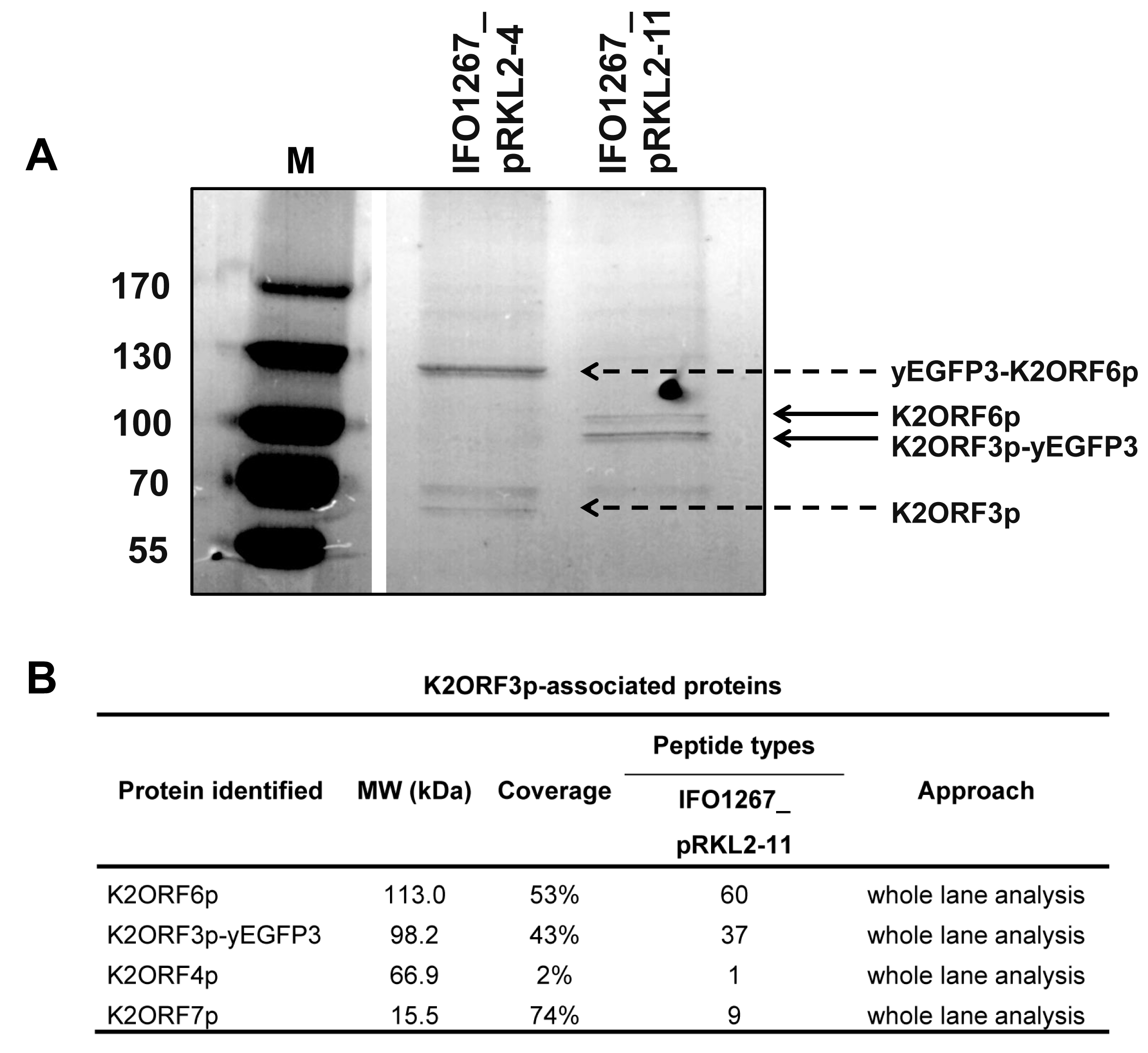

Supplement: S2 Fig — (A) IFO1267_pRKL2-4 (containing yEGFP3-K2ORF6p) and IFO1267_pRKL2-11 (K2ORF3p-yEGFP3) cells were grown to late exponential phase. The cells were lysed, yEGFP3-K2ORF6p and K2ORF3p-yEGFP3 were affinity-purified using GFP-Trap agarose beads. Bound proteins were digested with trypsin, and then analyzed by mass spectrometry. Also, an aliquot of the beads was taken and bound proteins were eluted and resolved by SDS-PAGE, and the gel was silver-stained. Proteins identified by mass spectrometry are indicated with arrows on the right side. Proteins identified by mass spectrometry in previous experiments described in Table 1 and Fig 1 are indicated with dashed arrows on the right side. Bands corresponding to small RNA polymerase subunit were not clearly visible, presumably due to their smaller mass and weaker staining. M, protein molecular mass marker (PageRuler Prestained Protein Ladder, Fermentas); the respective molecular mass values are indicated on the left side. (B) K2ORF3p-associated proteins identified by mass spectrometry. The proteins identified, their molecular weight (MW), unique coverage, and the number of peptide types from IFO1267_pRKL2-11 strain is listed. (TIF) [file ppat.1007377.s002.tif]

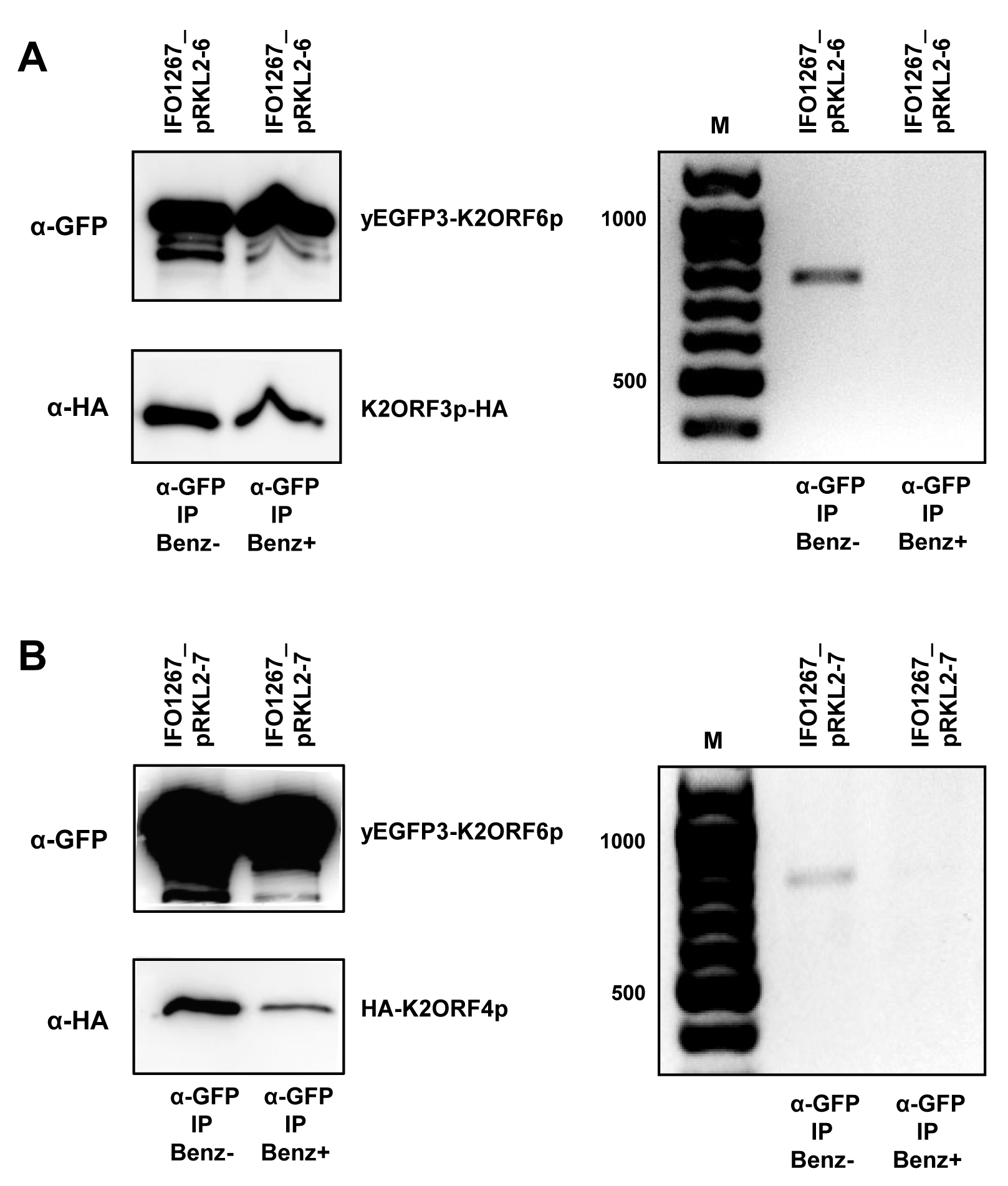

Supplement: S3 Fig — (A) yEGFP3-K2ORF6p was purified with GFP-Trap agarose beads from lysates of the IFO1267_pRKL2-6 strain cells. After washing the bound immunoprecipitated proteins (α-GFP IP), the beads were split into two parts which were mock-treated (Benz-) and treated (Benz+) with Benzonase Nuclease to digest DNA and RNA. Then, the beads were extensively washed again and the bound proteins were eluted and analysed by Western blotting using anti-GFP (α-GFP) and anti-HA (α-HA) antibodies. Also, an aliquot of the beads was taken and bound nucleic acids were eluted and analyzed for presence of pRKL2-6 VLE DNA by PCR amplification for 25 cycles and visualized by electrophoresis. M, DNA molecular mass marker (GeneRuler 100 bp Plus DNA Ladder, Fermentas); respective molecular mass values of two DNA fragments (500 bp and 1 000 bp) are indicated. (B) yEGFP3-K2ORF6p was purified with GFP-Trap agarose beads from lysates of IFO1267_pRKL2-7 strain cells. After washing the bound immunoprecipitated proteins (α-GFP IP), the beads were split into two parts that were mock-treated (Benz-) and treated (Benz+) with Benzonase Nuclease to digest DNA and RNA. Then, the beads were extensively washed again and the bound proteins were eluted and analyzed by Western blotting using anti-GFP (α-GFP) and anti-HA (α-HA) antibodies. Also, an aliquot of the beads was taken and bound nucleic acids were eluted and analyzed for presence of pRKL2-7 VLE DNA by PCR amplification for 22 cycles and electrophoresis. M, DNA molecular mass marker (GeneRuler 100 bp Plus DNA Ladder, Fermentas); respective molecular mass values of two DNA fragments (500 bp and 1 000 bp) are indicated. (TIF) [file ppat.1007377.s003.tif]

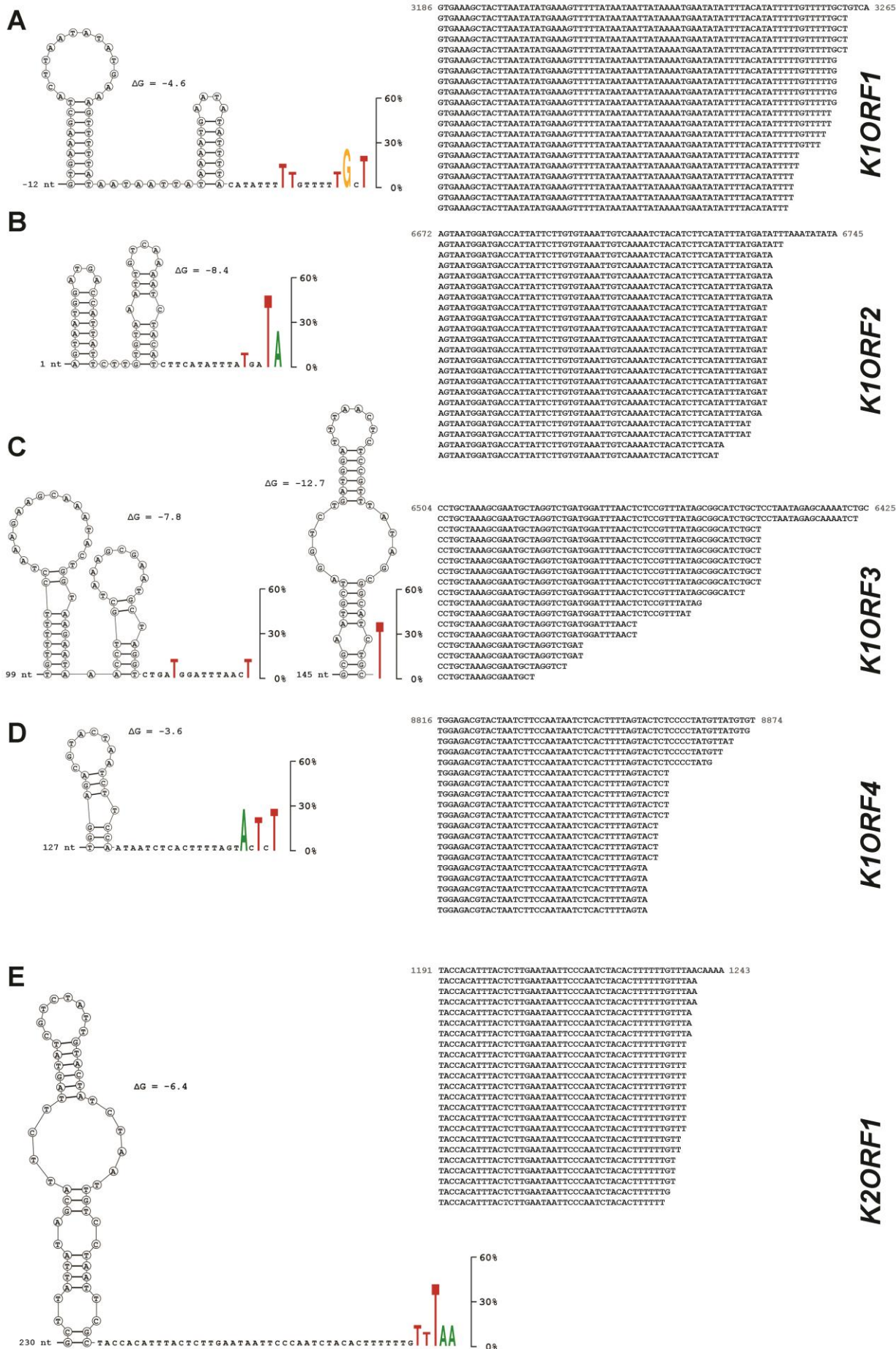





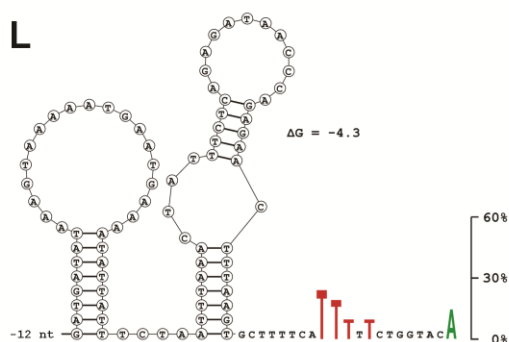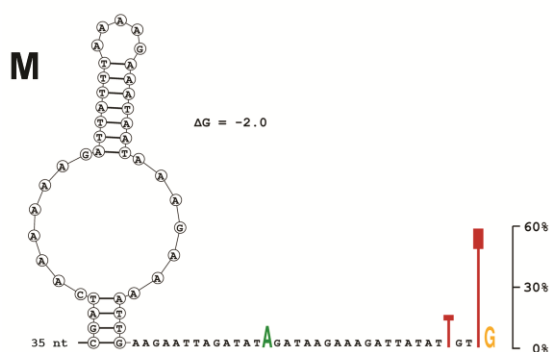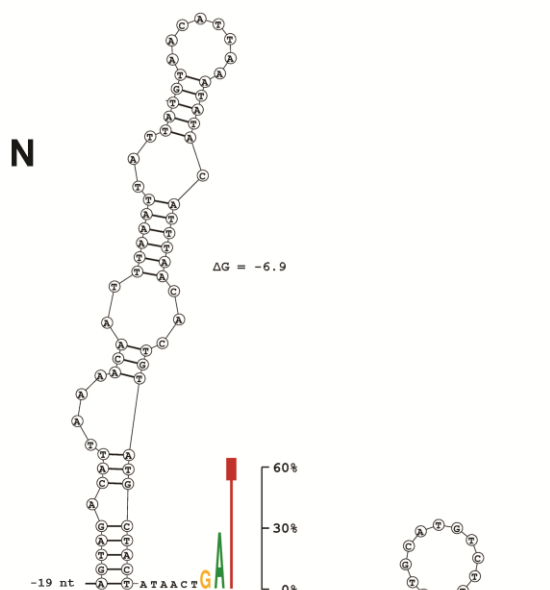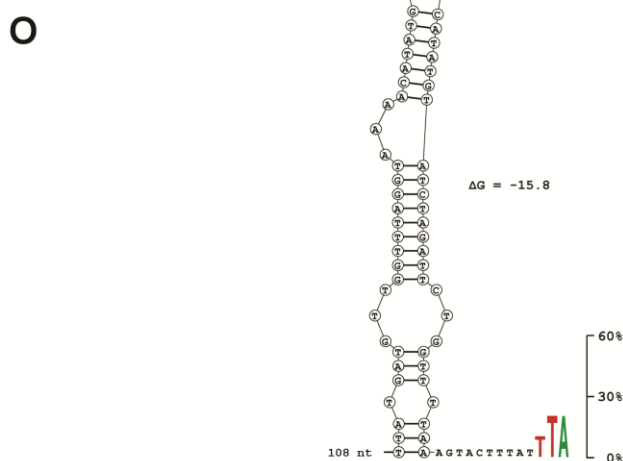[illegible][illegible][illegible][illegible]

**K2ORF8**

**K2ORF9**

**K2ORF10**

**K2ORF11**

Supplement: S4 Fig — This figure represents results of 3′ RACE-PCR analysis of individual mRNAs corresponding to all genes encoded by the pGKL elements. Total RNA was isolated from wild-type IFO1267 strain, DNase treated and 3′ polycytidinylated. Reverse transcription was carried out using oligo(dG)anch2 primer. Purified cDNA was used for 3′ RACE-PCR using anch2 primer and gene-specific primers listed in S2 Table. After PCR amplification and electrophoresis, the identity of the products was verified using restriction digestion and fragments exhibiting correct digestion pattern were gel-purified, cloned to the pCR4-TOPO vector and sequenced. The upper sequence on the right side corresponds to the template (plasmid) DNA and its position in the pGKL genome is annotated using K. lactis pGKL1 (X00762.1) and pGKL2 (X07776.1) sequences. Sequences situated below represent individual sequenced cDNA clones (only distal part of 3′ untranslated region is shown). Cytosine residues at the 3′ end of cDNA corresponding to the RNA tail are omitted in this representation for clarity. RNA secondary structures close to 3′ ends of VLE-specific mRNAs were predicted using default settings of RNAstructure Server (http://rna.urmc.rochester.edu/RNAstructureWeb/) [42]. Predicted RNA stem loops are displayed as cDNA nucleotide letters in circles on the left side, and the values of Gibbs free energy (ΔG) in kcal/mol are displayed for each structure. Stem loop distances from gene stop codon are shown as numbers of nucleotides (nt). Final nucleotides of the experimentally determined 3′ ends of cDNA are shown as colored letters enlarged proportionally to their occurrence (in percent) in the sequenced clones when the same final nucleotide was detected in at least two independent clones. Predicted RNA stem loop structures and sequenced cDNA clones are listed as follows: (A) K1ORF1, (B) K1ORF2, (C) K1ORF3, (D) K1ORF4, (E) K2ORF1, (F) K2ORF2, (G) K2ORF3, (H) K2ORF4, (I) K2ORF5, (J) K2ORF6, (K) K2ORF7, (L) K2ORF8, (M) K2OR [file ppat.1007377.s004.pdf]

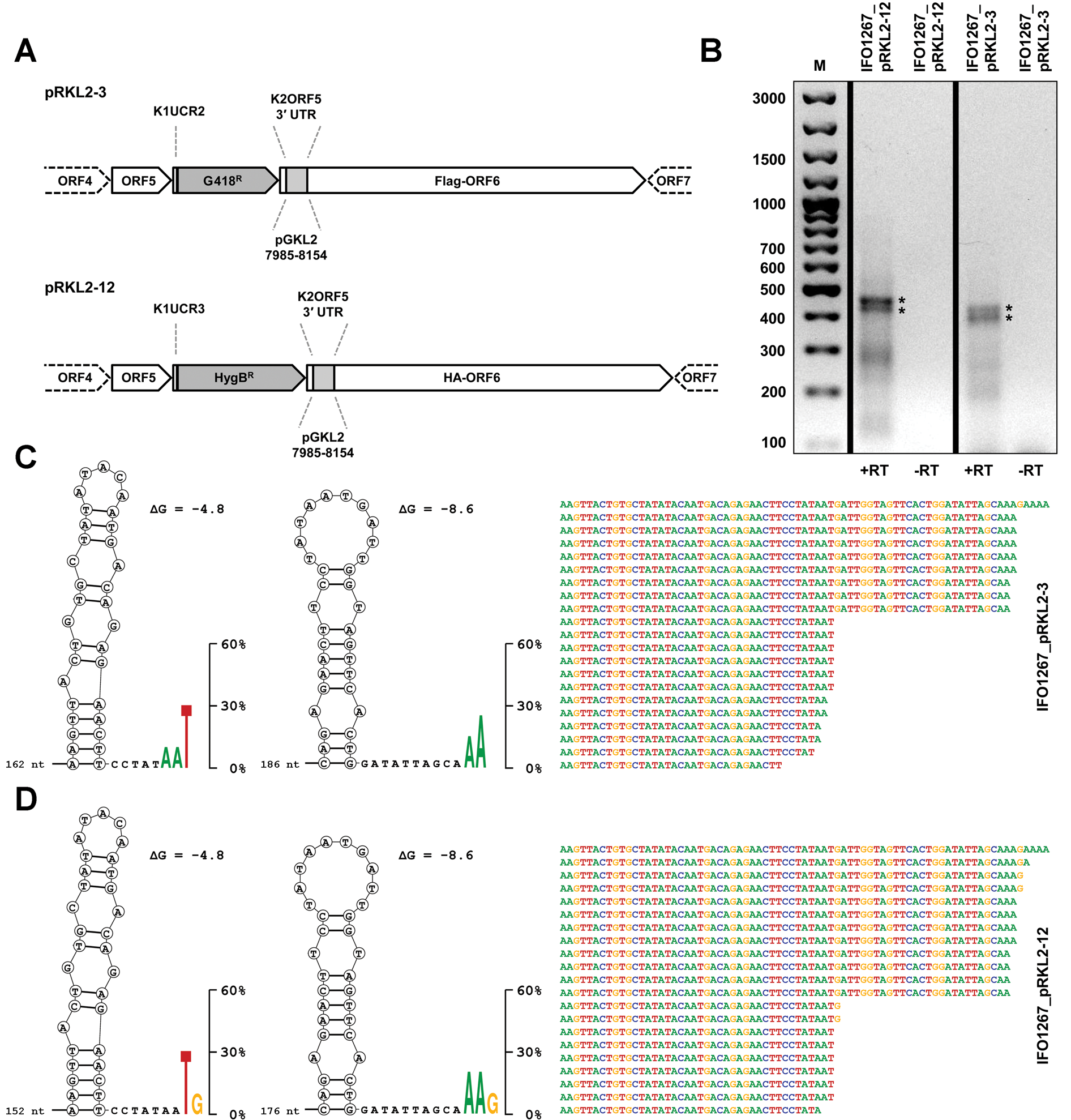

Supplement: S5 Fig — This figure represents results of 3′ RACE-PCR analysis of individual mRNAs corresponding to the G418 and hygromycin B resistance genes expressed under control of K1UCR2 and K1UCR3, respectively. (A) Schematic representation of recombinant pGKL2 elements where the resistance marker genes are inserted prior to K2ORF5 gene in the same transcriptional orientation. (B) Total RNA was isolated from IFO1267_pRKL2-3 and IFO1267_pRKL2-12 strains, DNase treated and 3′ polycytidinylated. Reverse transcription was carried out in the presence (+RT) and absence (-RT) of reverse transcriptase using oligo(dG)anch2 primer. Purified cDNA was used for 3′ RACE-PCR using anch2 primer and gene-specific primers listed in S2 Table. After PCR amplification the samples were analyzed in 1.8% agarose gel stained by ethidium bromide. M, DNA molecular mass marker (GeneRuler 100 bp Plus DNA Ladder, Fermentas). The respective values are indicated on the left side. Specific products that were cloned to the pCR4-TOPO vector and used for sequencing are labelled with asterisks. Predicted RNA stem loop structures and sequenced cDNA clones for mRNA 3′ ends of (C) G418 and (D) Hygromycin B resistance genes are depicted as in S4 Fig. (TIF) [file ppat.1007377.s005.tif]

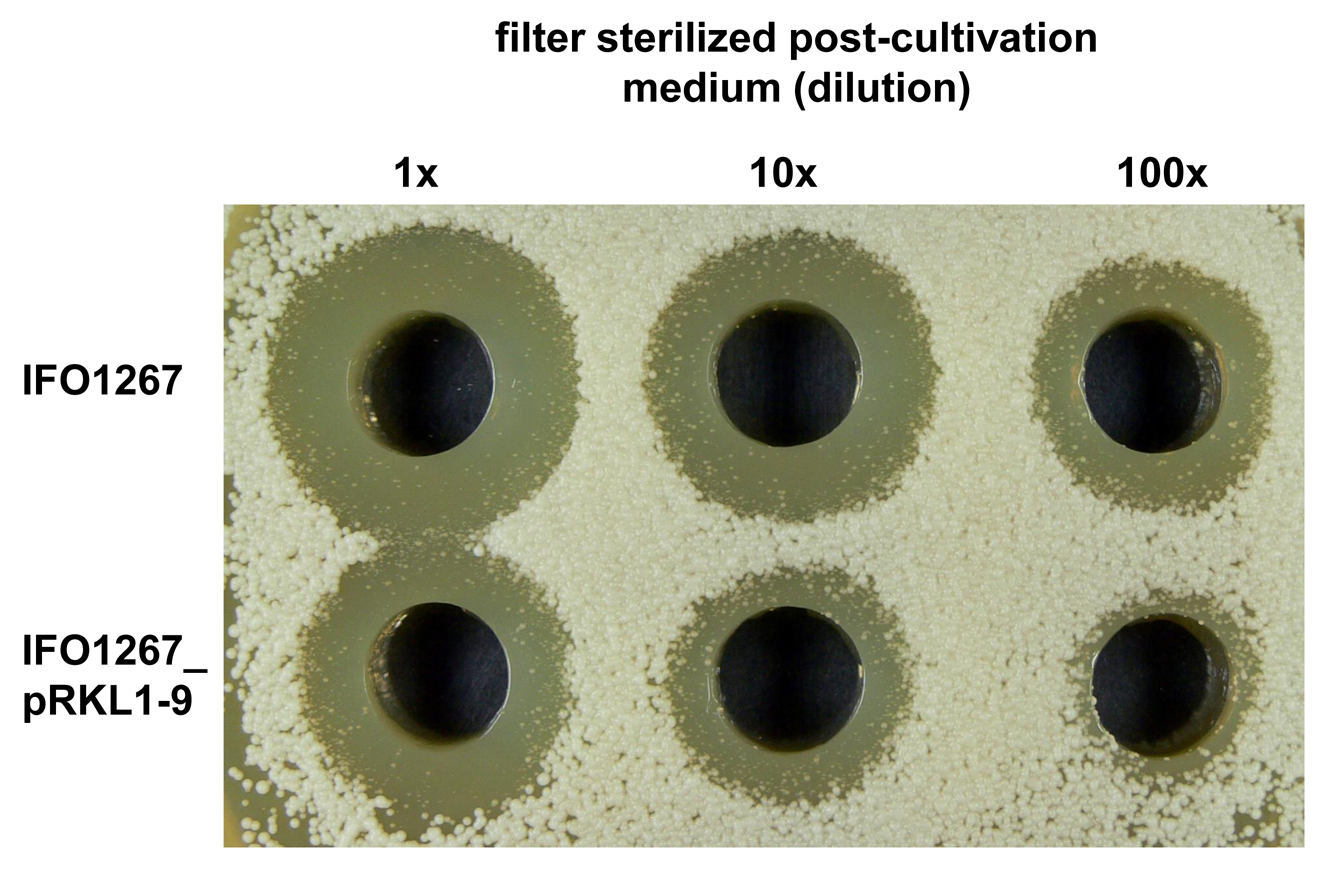

Supplement: S8 Fig — This figure represents results of killer toxin activity assay. IFO1267_pRKL1-9 (TACCC INR) and IFO1267 (control) cells were cultivated in YPD medium at 24°C. Aliquots were taken at 0, 3, 6 and 12 hours, and the culture medium was filter-sterilized, diluted, and assayed for the presence of the killer toxin activity by an agar well diffusion test using a lawn of S. cerevisiae S6/1 sensitive strain cells grown on YPD plates at 24°C for 2 days. Result from post-cultivation medium taken at 12 hours is shown. (TIF) [file ppat.1007377.s008.tif]
