# Supplementary material for: Transcription apparatus of the yeast virus-like elements: Architecture, function, and evolutionary origin
Source: PLoS Pathog. 2018 Oct 22;14(10):e1007377. doi: 10.1371/journal.ppat.1007377 (PMC6211774; doi:10.1371/journal.ppat.1007377)
Supplement: S6 Fig — Conserved regions are named according to ref. [46]. In the resulting sequence alignment the identity (black shading) was highlighted where the same amino acid residue occurred in ≥ 50% of the sequences and sequence similarity (gray shading) was highlighted where amino acid residue with similar properties occurred in ≥ 50% of the sequences. The numbers in brackets indicate the number of amino acid residues that were not displayed in this comparison. Sequence identity (in %) of consecutively numbered sequences is depicted in a table below each alignment. Local reliability of sequence alignments was evaluated using the Transitive Consistency Score (TCS) web server [88] and apart from the second half of βa6 conserved region alignment all alignments showed good or average local reliabilities. Following sequences (with their accession numbers) were used for the alignment: Thermus aquaticus β (CAB65465.2), Escherichia coli β (AAC76961.1) Methanocaldococcus jannaschii B′′ (Q58444.1), Methanocaldococcus jannaschii B′ (Q60181.1), Saccharomyces cerevisiae Rpa135 (CAA95050.1), Saccharomyces cerevisiae Rpb2 (NP_014794.1), Sascharomyces cerevisiae Rpc128 (CAA99422.1), Vaccinia virus Rpo132 (AAQ93241.1), Kluyveromyces lactis ORF6-pGKL2 (P05472.1), Saccharomyces kluyveri ORF6-pSKL (CAA38625.1), Pichia acaciae ORF6-pPac-1 (CAJ57280.1), Pichia etchellsii ORF6-pPE1B (CAC08226.1). (DOCX) [file ppat.1007377.s006.docx]

------ βa1 ------

T. aquaticus β 16 PPLTEIQVESYKKALQA
E. coli β 25 PYLLSIQLDSFQKFIEQ
M. jannaschii B’’ 15 HGLIDHQIESYNDFVEN
S. cerevisiae pol I Rpa135 39 QEAVQPHIGSFNALTEG
S. cerevisiae pol II Rpb2 41 KGLVSQQLDSFNQFVDY
S. cerevisiae pol III Rpc128 55 KGLVKQHLDSFNYFVDT
Vaccinia virus Rpo132 28 YRPLHFQYVSYSNFILH
K. lactis pGKL2 ORF6 2 ---DYGQIEIYNDYFRN
S. kluyveri pSKL ORF6 5 ---DYGQIKIYETLFED
P. acaciae pPac1-1 ORF6 5 ---DFGQIKIYEDLFHD
P. etchellsii pPE1B ORF6 5 ---NFGQIDIYNNIFSD

|  | [1] | [2] | [3] | [4] | [5] | [6] | [7] | [8] | [9] | [10] | [11] |
| --- | --- | --- | --- | --- | --- | --- | --- | --- | --- | --- | --- |
| T. aquaticus β [1] | 100% | 35% | 29% | 6% | 18% | 12% | 18% | 21% | 14% | 14% | 14% |
| E. coli β [2] | 35% | 100% | 29% | 18% | 47% | 35% | 29% | 7% | 14% | 7% | 14% |
| M. jannaschii B’’ [3] | 29% | 29% | 100% | 24% | 41% | 35% | 24% | 50% | 29% | 29% | 29% |
| S. cerevisiae pol I Rpa135 [4] | 6% | 18% | 24% | 100% | 24% | 29% | 6% | 14% | 21% | 14% | 14% |
| S. cerevisiae pol II Rpb2 [5] | 18% | 47% | 41% | 24% | 100% | 76% | 18% | 14% | 7% | 7% | 21% |
| S. cerevisiae pol III Rpc128 [6] | 12% | 35% | 35% | 29% | 76% | 100% | 12% | 7% | 0% | 0% | 14% |
| Vaccinia virus Rpo132 [7] | 18% | 29% | 24% | 6% | 18% | 12% | 100% | 14% | 14% | 14% | 21% |
| K. lactis pGKL2 ORF6 [8] | 21% | 7% | 50% | 14% | 14% | 7% | 14% | 100% | 57% | 57% | 50% |
| S. kluyveri pSKL ORF6 [9] | 14% | 14% | 29% | 21% | 7% | 0% | 14% | 57% | 100% | 79% | 50% |
| P. acaciae pPac1-1 ORF6 [10] | 14% | 7% | 29% | 14% | 7% | 0% | 14% | 57% | 79% | 100% | 57% |
| P. etchellsii pPE1B ORF6 [11] | 14% | 14% | 29% | 14% | 21% | 14% | 21% | 50% | 50% | 57% | 100% |

-------------- βa6 -------------

T. aquaticus β 328 LGNRRIRTVGELMADQFRVGLARLARGVRERM
E. coli β 448 LGNRRIRSVGEMAENQFRVGLVRVERAVKERL
M. jannaschii B’’ 339 YAYKRAKLAGDLMEDLFRYAFSQLVKDIKYQL
S. cerevisiae pol I Rpa135 397 TQHQEVLLGGFLYGMILKEKIDEYLQNIIAQV
S. cerevisiae pol II Rpb2 401 FGKKRLDLAGPLLAQLFKTLFKKLTKDIFRYM
S. cerevisiae pol III Rpc128 372 VGNKRLELAGQLISLLFEDLFKKFNNDFKLSI
Vaccinia virus Rpo132 354 MVCHRILTYGKYFETLAHDELENYIGNIRNDI
K. lactis pGKL2 ORF6 150 KAEKPIKNEIDLISLCYMFECWLGLREEPRLY
S. kluyveri pSKL ORF6 149 KSDKVIKNEIDLMIICYMFECWLGIKKESVHP
P. acaciae pPac1-1 ORF6 152 KTSNSIKNEVDLIHLCYMFECWFDLRKPPKNN
P. etchellsii pPE1B ORF6 153 KLNTAIKNEIDLINLCYMFECWLGYKQTKVLE

|  | [1] | [2] | [3] | [4] | [5] | [6] | [7] | [8] | [9] | [10] | [11] |
| --- | --- | --- | --- | --- | --- | --- | --- | --- | --- | --- | --- |
| T. aquaticus β [1] | 100% | 66% | 25% | 6% | 25% | 19% | 19% | 9% | 9% | 9% | 9% |
| E. coli β [2] | 66% | 100% | 22% | 3% | 13% | 19% | 16% | 6% | 3% | 6% | 9% |
| M. jannaschii B’’ [3] | 25% | 22% | 100% | 16% | 41% | 34% | 16% | 16% | 19% | 9% | 16% |
| S. cerevisiae pol I Rpa135 [4] | 6% | 3% | 16% | 100% | 16% | 9% | 16% | 6% | 3% | 6% | 3% |
| S. cerevisiae pol II Rpb2 [5] | 25% | 13% | 41% | 16% | 100% | 47% | 13% | 9% | 9% | 3% | 6% |
| S. cerevisiae pol III Rpc128 [6] | 19% | 19% | 34% | 9% | 47% | 100% | 16% | 16% | 6% | 9% | 16% |
| Vaccinia virus Rpo132 [7] | 19% | 16% | 16% | 16% | 13% | 16% | 100% | 6% | 9% | 6% | 6% |
| K. lactis pGKL2 ORF6 [8] | 9% | 6% | 16% | 6% | 9% | 16% | 6% | 100% | 59% | 59% | 63% |
| S. kluyveri pSKL ORF6 [9] | 9% | 3% | 19% | 3% | 9% | 6% | 9% | 59% | 100% | 47% | 59% |
| P. acaciae pPac1-1 ORF6 [10] | 9% | 6% | 9% | 6% | 3% | 9% | 6% | 59% | 47% | 100% | 50% |
| P. etchellsii pPE1B ORF6 [11] | 9% | 9% | 16% | 3% | 6% | 16% | 6% | 63% | 59% | 50% | 100% |

------- βa13 --------

T. aquaticus β 787 DTSLRVPPGEGGIVVGRLRLR
E. coli β 915 DSSLRVPNGVSGTVIDVQVFT
M. jannaschii B’ 324 DSSVVVRHGEEGYIDKVILTE
S. cerevisiae pol I Rpa135 869 TKIKTYHSSEPAYIEEVNLIG
S. cerevisiae pol II Rpb2 936 DASTPLRSTENGIVDQVLVTT
S. cerevisiae pol III Rpc128 868 EAPVIYRGPEPSHIDQVMMSV
Vaccinia virus Rpo132 841 DVSEKYTDMYKSRVERVQVEL
K. lactis pGKL2 ORF6 470 KTINFVYAEHDGKVKEIIREE
S. kluyveri pSKL ORF6 472 KTINFVYAEHDGKVKEIIREE
P. acaciae pPac1-1 ORF6 474 KTINFVYAEHDGKVKEIIREE
P. etchellsii pPE1B ORF6 475 KTINFVYAEHDGKVKEIIREE

|  | [1] | [2] | [3] | [4] | [5] | [6] | [7] | [8] | [9] | [10] | [11] |
| --- | --- | --- | --- | --- | --- | --- | --- | --- | --- | --- | --- |
| T. aquaticus β [1] | 100% | 43% | 29% | 5% | 33% | 5% | 14% | 24% | 24% | 24% | 24% |
| E. coli β [2] | 43% | 100% | 33% | 5% | 33% | 5% | 29% | 14% | 14% | 14% | 14% |
| M. jannaschii B’’ [3] | 29% | 33% | 100% | 24% | 38% | 29% | 14% | 19% | 19% | 19% | 19% |
| S. cerevisiae pol I Rpa135 [4] | 5% | 5% | 24% | 100% | 14% | 24% | 14% | 10% | 10% | 10% | 10% |
| S. cerevisiae pol II Rpb2 [5] | 33% | 33% | 38% | 14% | 100% | 29% | 24% | 10% | 10% | 10% | 10% |
| S. cerevisiae pol III Rpc128 [6] | 5% | 5% | 29% | 24% | 29% | 100% | 14% | 0% | 0% | 0% | 0% |
| Vaccinia virus Rpo132 [7] | 14% | 29% | 14% | 14% | 24% | 14% | 100% | 10% | 10% | 10% | 10% |
| K. lactis pGKL2 ORF6 [8] | 24% | 14% | 19% | 10% | 10% | 0% | 10% | 100% | 100% | 100% | 100% |
| S. kluyveri pSKL ORF6 [9] | 24% | 14% | 19% | 10% | 10% | 0% | 10% | 100% | 100% | 100% | 100% |
| P. acaciae pPac1-1 ORF6 [10] | 24% | 14% | 19% | 10% | 10% | 0% | 10% | 100% | 100% | 100% | 100% |
| P. etchellsii pPE1B ORF6 [11] | 24% | 14% | 19% | 10% | 10% | 0% | 10% | 100% | 100% | 100% | 100% |

SWITCH 3 and 4 --------- CLAMP ---------

------- βa15 ------ -------- βa16 -------

T. aquaticus β 1053 LTIKSDDIEGRNAAYQAII.[ 8].SVPESFRVLVKELQALALDVQTLDE
E. coli β 1291 LTVKSDDVNGRTKMYKNIV.[ 8].GMPESFNVLLKEIRSLGINIELEDE
M. jannaschii B’ 539 LMDESDPYDICICSKCGDF.[30].RIPYAFKLLLDELKSMCILPRIKVR
S. cerevisiae pol I Rpa135 1092 LLNSSDYTQASVCRECGSI.[66].AIPFVLKYLDSELSAMGIRLRYNVE
S. cerevisiae pol II Rpb2 1151 LMEASDAFRVHICGICGLM.[25].HIPYAAKLLFQELMAMNITPRLYTD
S. cerevisiae pol III Rpc128 1083 LMISSDAFEVDVCDKCGLM.[18].TIPYAAKLLFQELLSMNIAPRLRLE
Vaccinia virus Rpo132 1074 LKDSEEYQDVYVCENCGDI.[25].DTTHVSKVFLTQMNARGVKVKLDFE
K. lactis pGKL2 ORF6 643 W--SIDRSKIKIDSKTGRI.[ 9].EIGQYFKICLSYMRSLGRDILIKND
S. kluyveri pSKL ORF6 647 W--SIDKTNIYVCPKSGKI.[ 9].ESHQYFKICLSYMRALGHDILIKNN
P. acaciae pPac1-1 ORF6 650 W--NIDKTITYCCPKSGII.[ 9].EVNQYLLICLSILRALDYDIQIKDN

P. etchellsii pPE1B ORF6 650 W--SVDRSIIKVCPKTGII.[11].EINQGFIICLSYLRGLGYDIRLKNN

|  | [1] | [2] | [3] | [4] | [5] | [6] | [7] | [8] | [9] | [10] | [11] |
| --- | --- | --- | --- | --- | --- | --- | --- | --- | --- | --- | --- |
| T. aquaticus β [1] | 100% | 48% | 18% | 23% | 18% | 23% | 14% | 12% | 14% | 24% | 17% |
| E. coli β [2] | 48% | 100% | 23% | 20% | 18% | 23% | 14% | 19% | 19% | 17% | 21% |
| M. jannaschii B’’ [3] | 18% | 23% | 100% | 36% | 48% | 50% | 20% | 31% | 24% | 19% | 26% |
| S. cerevisiae pol I Rpa135 [4] | 23% | 20% | 36% | 100% | 36% | 41% | 30% | 19% | 26% | 21% | 26% |
| S. cerevisiae pol II Rpb2 [5] | 18% | 18% | 48% | 36% | 100% | 68% | 18% | 14% | 12% | 12% | 17% |
| S. cerevisiae pol III Rpc128 [6] | 23% | 23% | 50% | 41% | 68% | 100% | 23% | 17% | 17% | 12% | 24% |
| Vaccinia virus Rpo132 [7] | 14% | 14% | 20% | 30% | 18% | 23% | 100% | 17% | 26% | 14% | 19% |
| K. lactis pGKL2 ORF6 [8] | 12% | 19% | 31% | 19% | 14% | 17% | 17% | 100% | 67% | 45% | 64% |
| S. kluyveri pSKL ORF6 [9] | 14% | 19% | 24% | 26% | 12% | 17% | 26% | 67% | 100% | 64% | 62% |
| P. acaciae pPac1-1 ORF6 [10] | 24% | 17% | 19% | 21% | 12% | 12% | 14% | 45% | 64% | 100% | 57% |
| P. etchellsii pPE1B ORF6 [11] | 17% | 21% | 26% | 26% | 17% | 24% | 19% | 64% | 62% | 57% | 100% |
