# Supplementary material for: Transcription apparatus of the yeast virus-like elements: Architecture, function, and evolutionary origin
Source: PLoS Pathog. 2018 Oct 22;14(10):e1007377. doi: 10.1371/journal.ppat.1007377 (PMC6211774; doi:10.1371/journal.ppat.1007377)
Supplement: S1 Table — (DOCX) [file ppat.1007377.s010.docx]

| **Plasmid / Element** | **Size (bp)** | **Description of relevant features** | **Reference / Source** |
| --- | --- | --- | --- |
| pGKL1 | 8874 | native, kil+ | GenBank: X00762.1 |
| pGKL2 | 13457 | native | GenBank:  X07776.1 |
| pRKL1-1 | 6425 | modified pGKL1 containing G418 resistance marker under control of UCR2 from pGKL1 plasmid (K1UCR2), deleted ORF2 gene, kil- | This study |
| pRKL1-2 | 6425 | modified pGKL1 containing G418 resistance marker under control of K1UCR2 with one point mutation in putative initiator region, deleted ORF2 gene, kil- | This study |
| pRKL1-3 | 6425 | modified pGKL1 containing G418 resistance marker under control of K1UCR2 with two point mutations in putative initiator region, deleted ORF2 gene, kil- | This study |
| pRKL1-4 | 9970 | modified pGKL1 containing ORF4-HA gene under control of K1UCR4 and hygromycin B resistance marker under control of K2UCR6 | This study |
| pRKL1-5 | 6595 | modified pGKL1 containing G418 resistance marker under control of K1UCR2 followed by 3′ untranslated region of K2ORF5 gene, deleted ORF2 gene, kil- | This study |
| pRKL1-6 | 6595 | modified pGKL1 containing G418 resistance marker under control of K1UCR2 followed by 3′ untranslated region of K2ORF5 gene with mutations in putative Stem loop 2, deleted ORF2 gene, kil- | This study |
| pRKL1-7 | 6595 | modified pGKL1 containing G418 resistance marker under control of K1UCR2 followed by 3′ untranslated region of K2ORF5 gene with rescue mutations in putative Stem loop 2, deleted ORF2 gene, kil- | This study |
| pRKL1-9 | 9988 | modified pGKL1 containing K1ORF4 under control of K1UCR4 with two point mutations in putative initiator region and hygromycin B resistance marker under control of K2UCR6 | This study |
| pRKL2-3 | 14368 | modified pGKL2 containing FLAG-ORF6 gene under control of K2UCR6 and G418 resistance marker under control of K1UCR2 | This study |
| pRKL2-4 | 15082 | modified pGKL2 containing fusion yEGFP3-ORF6 gene under control of K2UCR6 and G418 resistance marker under control of K1UCR2 | This study |
| pRKL2-5 | 16199 | modified pRKL2-4 containing ORF7-FLAG gene under control of K2UCR7 and hygromycin B resistance marker under control of K1UCR1 | This study |
| pRKL2-6 | 16206 | modified pRKL2-4 containing ORF3-HA gene under control of K2UCR3, ORF2 gene under control of K1UCR1 and hygromycin B resistance marker under control of K1UCR3 | This study |
| pRKL2-7 | 16213 | modified pRKL2-4 containing HA-ORF4 gene under control of K2UCR4 and hygromycin B resistance marker under control of K1UCR3 | This study |
| pRKL2-8 | 15104 | modified pGKL2 containing fusion yEGFP3-ORF4 gene under control of K2UCR4 and G418 resistance marker under control of K1UCR2 | This study |
| pRKL2-9 | 16194 | modified pRKL2-8 containing HA-ORF6 gene under control of K2UCR6 and hygromycin B resistance marker under control of K1UCR3 | This study |
| pRKL2-10 | 16228 | modified pRKL2-8 containing ORF3-HA gene under control of K2UCR3, ORF2 gene under control of K1UCR1 and hygromycin B resistance marker under control of K1UCR3 | This study |
| pRKL2-11 | 15094 | modified pGKL2 containing fusion ORF3-yEGFP3 gene under control of K2UCR3, ORF2 gene under control of K1UCR1 and G418 resistance marker under control of K1UCR2 | This study |
| pRKL2-12 | 14547 | modified pGKL2 containing HA-ORF6 gene under control of K2UCR6 and hygromycin B resistance marker under control of K1UCR3 | This study |
| pRKL2-13 | 14588 | modified pGKL2 containing HA-ORF4 gene under control of K2UCR4 and hygromycin B resistance marker under control of K1UCR3 | This study |
| pRKL2-14 | 14581 | modified pGKL2 containing ORF3-HA gene under control of K2UCR3, ORF2 gene under control of K1UCR1 and hygromycin B resistance marker under control of K1UCR3 | This study |
| pRKL2-15 | 14574 | modified pGKL2 containing ORF7-FLAG gene under control of K2UCR7 and hygromycin B resistance marker under control of K1UCR1 | This study |
| pUG6 | 4009 | β-lactamase, colE1 *ori*, loxP-G418 resistance marker-loxP | GenBank:  AF298793.1 |
| pUG36 | 6225 | CEN6/ARSH4, URA3, β-lactamase, colE1 *ori*, MET25 promoter, yEGFP3 | GenBank: AF298791.1 |
| pcDNA5/FRT/TO | 5137 | β-lactamase, pUC *ori*, CMV promoter/Tetracycline operator, FRT, hygromycin B resistence | Invitrogen |
| pCR4-TOPO | 3956 | β-lactamase, aph(3')-II, pUC *ori*, pLac, lacZα-ccdB | Invitrogen |
