# Supplementary material for: Transcription apparatus of the yeast virus-like elements: Architecture, function, and evolutionary origin
Source: PLoS Pathog. 2018 Oct 22;14(10):e1007377. doi: 10.1371/journal.ppat.1007377 (PMC6211774; doi:10.1371/journal.ppat.1007377)
Supplement: S2 Table — (DOCX) [file ppat.1007377.s011.docx]

| **ORF / gene** | **Primer name** | **Sequence (5′-3′)** | **Used in** | **Related to Figure** |
| --- | --- | --- | --- | --- |
| K1UCR2 + G418 (5′ end) | pGKL-kanF | ATAAGACCGTGAAAGCTACTTAATATATGAAAGTTTTTATAATAATTATAAAATGGGTAAGGAAAAGACTCACG | pRKL1-1/pRKL1-5/pRKL1-6/pRKL1-7 construction | 4, 5, S1 |
| G418 (3′ end) + K1ORF2 (3′ end) | pGKL-kanR | GTTTTTCCTAAAGAAGCAAATACTGGTAAGAATAAACCTGCTAAAGCGAATGTTAGAAAAACTCATCGAGCATCA | pRKL1-1/pRKL1-2/pRKL1-3 construction | 4, S1 |
| K1UCR2* + G418 (5′ end) | K1O2_prom_del_F | ATAAGACCGTGAAAGCTACTTAATATATGAAAGTTTTTATAATAATTATAACATGGGTAAGGAAAAGACTCACG | pRKL1-2 construction | 4, S1 |
| K1UCR2** + G418 (5′ end) | K1O2_prom_del2_F | ATAAGACCGTGAAAGCTACTTAATATATGAAAGTTTTTATAATAATTATACCATGGGTAAGGAAAAGACTCACG | pRKL1-3 construction | 4, S1 |
| K1ORF4 | K07 | AATGAAGATATATCATATATTTAG | pRKL1-4 construction | 2, S1 |
| K1ORF4-HA (3′ end) + K2UCR6 | K1_ORF4-HA_R1 | TTTAGGTATATCTAATACGATCATATATCAAGCATAATCTGGAACATCATATGGATATACACATTTTCCATTCTGTAGA | pRKL1-4 construction | 2, S1 |
| K2UCR6 + HygB (5′ end) | K1_ORF4-HA_F2 | TATATGATCGTATTAGATATACCTAAAATGAAAAAGCCTGAACTCACCGCGAC | pRKL1-4 construction | 2, S1 |
| HygB (3′ end) + K1 TIR | K1_ORF4-HA_R2 | GTCTATTTTACACTTTTGACCTATAAGTCATTTTATTATACACATTTTCCATTATTCCTTTGCCCTCGGACGAGTG | pRKL1-4 construction | 2, S1 |
| G418 (3′ end) | kanR2 | TTAGAAAAACTCATCGAGCATCA | pRKL1-5/pRKL1-6/pRKL1-7/pRKL2-8 construction | 2, 5, S1 |
| G418 (3′ end) + K2ORF5 3′ UTR (5′ end) | K1_K2O5-UTR_2F | CATTTGATGCTCGATGAGTTTTTCTAAGATTATGGACAAATAGAAATTT | pRKL1-5/pRKL1-6/pRKL1-7 construction | 5, S1 |
| K2ORF5 3′ UTR (3′ end) + K1ORF2 (3′ end) | K1_K2O5-UTR_2R | GTTTTTCCTAAAGAAGCAAATACTGGTAAGAATAAACCTGCTAAAGCGAATGTTTTCTTTGCTAATATCCAGTG | pRKL1-5 construction | 5, S1 |
| K2ORF5 3′ UTR° (3′ end) + K1ORF2 (3′ end) | K1_K2O5-UTRm_2R | GTTTTTCCTAAAGAAGCAAATACTGGTAAGAATAAACCTGCTAAAGCGAATGTTTTCTTTGCTAATATCCAGTCGTGTACCAATCATTATAGGAAGTTC | pRKL1-6 construction | 5, S1 |
| K2ORF5 3′ UTR°° (3′ end) + K1ORF2 (3′ end) | K1_K2O5-UTRres_2R | GAAGCAAATACTGGTAAGAATAAACCTGCTAAAGCGAATGTTTTCTTTGCTAATATCCAGTCGTGTACCAATCATTATAGGAACACGTCTGTCATTGTATATAGCAC | pRKL1-7 construction | 5, S1 |
| K1ORF3 (3′ end) | 5RACE_O3_K1_2 | GTATCTTCTTCGCAAAAAGATCTTC | pRKL1-9 construction | S8 |
| K1ORF3 (5′ end) + K1UCR3 | K1_UCRmut-O4_R1 | ATATGATATATCTTCATTTTAATTAAAAATGTGTTTAGAATTAGATAATAATC | pRKL1-9 construction | S8 |
| K1UCR3 + K2UCR6 + HygB (5′ end) | K1_HA-ORF3_F2 | TTTTAATTAAAATGAAGATATATCATATTATATGATCGTATTAGATATACCTAAAATGAAAAAGCCTGAACTCACCGCGAC | pRKL1-9 construction | S8 |
| HygB (3′ end) + K1UCR4** + K1ORF4 (5′ end) | K1_UCRmut-O4_R2 | AAACACTAAATATATGATATATCTTCATTTTAATTAAAAATGTGGGTAGAATTAGATAATAATCTTTCAGATTTATTCCTTTGCCCTCGGACGAGTG | pRKL1-9 construction | S8 |
| K2ORF5 (3′ end) + K1UCR2 | KL_orf6N_Flag1F | AGAAGTTGAATTTGACAAACTATCATTAGCTATATGATCGTATTAGGACCGTGAAAGCTACTTAATATATGAAAG | pRKL2-3/pRKL2-4 construction | 1, 2, S1, S2, S5 |
| G418 (3′ end) + K2UCR6 + FLAG (5′ end) | KL_orf6N_Flag1R | CATCTTTATAATCCATTTTAGGTATATCTAATACGATCATATAGCTAATGATATTAGAAAAACTCATCGAGCATCAA | pRKL2-3 construction | S1, S5 |
| K2UCR6 + FLAG-K2ORF6 (5′ end) | KL_orf6N_Flag2F | TATATGATCGTATTAGATATACCTAAAATGGATTATAAAGATGATGATGATAAAATTGATTATGGACAAATAGAAATTTA | pRKL2-3 construction | S1,S5 |
| K2ORF6 | in_ORF6_rev | TCTCCTTTCTTTCATAGGTGC | pRKL2-3/pRKL2-9/pRKL2-12 construction / pRKL2-3 verification | 2, S1, S5 |
| G418 (3′ end) + K2UCR6 + yEGFP3 (5′ end) | KL_orf6N_EGFP1R | CTTCACCTTTAGACATTTTAGGTATATCTAATACGATCATATAGCTAATGATATTAGAAAAACTCATCGAGCATCAA | pRKL2-4 construction | 1, 2, S1, S2 |
| K2UCR6 + yEGFP3 (5′ end) | KL_orf6N_EGFP2F | TATATGATCGTATTAGATATACCTAAAATGTCTAAAGGTGAAGAATTATTCACTGGTG | pRKL2-4 construction | 1, 2, S1, S2 |
| yEGFP3 (3′ end) + K2ORF6 (5′ end) | KL_orf6N_EGFP2R | CAACATTTCTAAAATAATCGTTATAAATTTCTATTTGTCCATAATCCTGCAGCCCGGGGGATCCACTAGTT | pRKL2-4 construction | 1, 2, S1, S2 |
| K2ORF6 (3′ end) + K2ORF7-FLAG (3' end) | KL_orf7C_Flag1F | TTAGAAAATATATGTAATGAAATGTACTTATAATTATTTATCATCATCATCTTTATAATCATTTAAAAAGAATATTTTCTCGCTAGGGT | pRKL2-5/pRKL2-15 construction | 2, S1 |
| K2ORF7 (5′ end) + K2UCR7 + HygB (3' end) | KL_orf7C_Flag1R | AGCACTCGTCCGAGGGCAAAGGAATAATGTGAAGTTGATGATATAAAGTAAAAATGAATGAAAATATTATTTCTAA | pRKL2-5/pRKL2-15 construction | 2, S1 |
| HygB (3′ end) | KL_orf7C_Flag2F | TTATTCCTTTGCCCTCGGACGAGTG | pRKL2-5/pRKL2-15 construction | 2, S1 |
| HygB (5′ end) + K1UCR1 + K2ORF8 (3′ end) | KL_orf7C_Flag2R | TTATGTGAAGTTGATGATATAAAGTAAACTATAATATATGAATTACATTATTAATTTAAAAATGAAAAAGCCTGAACTCACCGCGAC | pRKL2-5/pRKL2-15 construction / pRKL2-5/pRKL2-15 verification | 2, S1 |
| K2ORF2 (5′ end) + K1UCR1 + HygB (3' end) | pGKL_ORF3-HA_F1 | TTAAGAATGCTAATTCATCATTCATTTTTAAATTAATAATGTAATTCATATATTATAGTTTATTCCTTTGCCCTCGGACGAGTG | pRKL2-6/pRKL2-10/pRKL2-14 construction / verification | 2, S1 |
| HygB (5′ end) + K1UCR3 | pGKL_ORF3-HA_R1 | ATATATGATATATCTTCATTTTAATTAAAAATGAAAAAGCCTGAACTCACCGCGAC | pRKL2-6/pRKL2-10/pRKL2-14 construction / verification | 2, S1 |
| K1UCR1 + K2ORF3-HA (3′ end) | pGKL_ORF3-HA_F2 | TTTTAATTAAAATGAAGATATATCATATATTCAAGCATAATCTGGAACATCATATGGATATCCTTTTTTAGAAAAGAAATGATAAG | pRKL2-6/pRKL2-10/pRKL2-14 construction | 2, S1 |
| K2ORF3 | K2_ORF3_for_seq | ATGAAGAATTTTCAGAAAGAAGTCC | pRKL2-6/pRKL2-10/pRKL2-14 construction | 2, S1 |
| K2ORF11 (3′ end) + K1UCR3 + HygB (5′ end) | pGKL_HA-ORF4_F1 | AACAATCAGATTAAAAATGAAAGTATTCACTAAATATATGATATATCTTCATTTTAATTAAAAATGAAAAAGCCTGAACTCACCGCGAC | pRKL2-7/pRKL2-13 construction / verification | 2, 3, S1 |
| HygB (3′ end) + K2UCR4 | pGKL_HA-ORF4_R1 | TTTTAATCTGATTGTTATAATCAGATAGTTATTCCTTTGCCCTCGGACGAGTG | pRKL2-7/pRKL2-13 construction / verification | 2, 3, S1 |
| K2UCR4 + HA-K2ORF4 (5′ end) | pGKL_HA-ORF4_F2 | CTATCTGATTATAACAATCAGATTAAAAATGTATCCATATGATGTTCCAGATTATGCTGGTAAAGTATTCACTAATAAAAAT | pRKL2-7/pRKL2-13 construction | 2, 3, S1 |
| K2ORF4 | 5RACE_O4_K2 | ATCTAGAATCAAGAACAACTTTCTCA | pRKL2-7/pRKL2-13 construction / pRKL2-7/pRKL2-8/pRKL2-10/pRKL2-13 verification / pRKL2-7/pRKL2-8/pRKL2-10 sequencing | 2, 3, S1 |
| K2ORF11 (3′ end) + K1UCR2 | EGFP-ORF4_1F | GATTATAACAATCAGATTAAAAATGAAAGTATTCACTAAGACCGTGAAAGCTACTTAATATATG | pRKL2-8 construction | 2, S1 |
| G418 (3′ end) + K2UCR4 + yEGFP3 (5′ end) | EGFP-ORF4_2F | GTTTCATTTGATGCTCGATGAGTTTTTCTAACTATCTGATTATAACAATCAGATTAAAAATGTCTAAAGGTGAAGAATTATTCACTGGTG | pRKL2-8 construction | 2, S1 |
| yEGFP3 (3′ end) + K2ORF4 (5′ end) | EGFP-ORF4_2R | GTATTGTTCTATACTTAAATTTTTATTAGTGAATACTTTCATCTGCAGCCCGGGGGATCCACTAGTT | pRKL2-8 construction | 2, S1 |
| K2ORF5 (3′ end) + K1UCR3 + HygB (5′ end) | pGKL_HA-ORF6_F1 | GAAGTTGAATTTGACAAACTATCATTAGCTATATGAATATATGATATATCTTCATTTTAATTAAAAATGAAAAAGCCTGAACTCACCGCGAC | pRKL2-9/pRKL2-12 construction / verification | 2, S1, S5 |
| HygB (3′ end) + K2UCR6 | pGKL_HA-ORF6_R1 | TTTAGGTATATCTAATACGATCATATATTATTCCTTTGCCCTCGGACGAGTG | pRKL2-9/pRKL2-12 construction | 2, S1, S5 |
| K2UCR6 + HA-K2ORF6 (5′ end) | pGKL_HA-ORF6_F2 | TATATGATCGTATTAGATATACCTAAAATGTATCCATATGATGTTCCAGATTATGCTGATTATGGACAAATAGAAATTTA | pRKL2-9/pRKL2-12 construction | 2, S1, S5 |
| K2ORF2 (3′ end) + K1UCR1 + G418 (3′ end) | pGKL2_ORF3-EGFP_F1 | CATCTATTTGAGAATTTAAGAATGCTAATTCATCATTCATTTTTAAATTAATAATGTAATTCATATATTATAGTTTAGAAAAACTCATCGAGCATC | pRKL2-11 construction | S1, S2 |
| K1UCR2 | KL_orf6C_Flag2F | GACCGTGAAAGCTACTTAATATATGAAAGTTTTT | pRKL2-11 construction | S1, S2 |
| K1UCR2 + yEGFP3 (3′ end) | pGKL2_ORF3-EGFP_F2 | TTCATATATTAAGTAGCTTTCACGGTCTCACTGCAGCCCGGGGGATCCAC | pRKL2-11 construction | S1, S2 |
| yEGFP3 (5′ end) + K2ORF4 (3′ end) | pGKL2_ORF3-EGFP_R2 | CAAGAAATAGAATTATCACGCATGTATTCTTATCATTTCTTTTCTAAAAAATCTAAAGGTGAAGAATTATTC | pRKL2-11 construction | S1, S2 |
| G418 | in_Kan_rev1 | GCAGTGGTGAGTAACCATGCA | 5´ RACE / pRKL1-1/pRKL1-2/ pRKL1-3/pRKL2-11 verification / sequencing | 2, 4, S1, S2 |
| G418 | KanVerF | AGACCGATACCAGGATCTTGCCATC | 3´ RACE / pRKL1-5/pRKL1-6/pRKL1-7/pRKL2-8/pRKL2-10/pRKL2-11 verification / pRKL1-5/pRKL1-6/pRKL1-7/pRKL2-8/pRKL2-10 sequencing | 2, 5, S1, S2, S4, S9 |
| G418 | KanR1 | ATGGGTAAGGAAAAGACTCACG | pRKL2-3/pRKL2-4 verification / pRKL2-4 sequencing | 1, 2, S1, S2, S5 |
| HygB | Hygro_rev | CGGAGACGCTGTCGAACT | pRKL1-4/pRKL2-9/pRKL2-12 verification / pRKL1-4 sequencing | 2, S1, S5 |
| HygB | Hygro_tail | ACGGCAATTTCGATGATGCAG | 3´ RACE / pRKL1-9 verification | S5, S8 |
| universal | olig2(dC)anchor | GACCACGCGTATCGATGTCGACCCCCCCCCCCC | 5´ RACE | 4 |
| universal | oligo(dG)anch2 | GATTGAGGTGTATCTGATGTCGAGGGGGGGGGGGG | 3´ RACE | 5, S4, S5, S9 |
| universal | anch2 | GATTGAGGTGTATCTGATGTCGA | 3´ RACE | 5, S4, S5, S9 |
| FLAG | FLAG-rev | TCTTTATCATCATCATCTTTATAATC | pRKL2-5/pRKL2-15 verification | 2, S1 |
| ACT | actin_KL-RNAfor | TGGTATGTGTAAAGCCGGTT | ChIP | 3 |
| ACT | aktin_KL-rev | AACACCGTCACCAGAATCCAA | ChIP | 3 |
| HGT1 | HGT1_KL-forw | GTTCGGTTTTGATATCGCATC | ChIP | 3 |
| HGT1 | HGT1_KL-rev | TGACAACCGTAACCGATGTAG | ChIP | 3 |
| K1ORF1 | ORF1-K1_tail_2 | TGGGACACTGATTTTATGCTG | pRKL1-1/pRKL1-2/ pRKL1-3 verification | 4, S1 |
| K1ORF1 | ORF1-K1_tail_3 | AGGATCAGAAGTAGGACAATTAGAAT | 3´ RACE | S4, S9 |
| K1ORF2 | ORF2-pGKL1_tail | TGAGGTCTTAGAAAGTGTGGTTG | 3´ RACE | S4, S9 |
| K1ORF3 | K05 | AAAATGTGTTTAGAATTAGA | ChIP | 3 |
| K1ORF3 | 5RACE_O3_K1_3 | TAGGATACCAAATTCCTGAAGGC | ChIP | 3 |
| K1ORF3 | ORF3-pGKL1_tail | ACTTATCGATTTTCCACGCC | pRKL1-5/pRKL1-6/pRKL1-7 verification | 5, S1 |
| K1ORF3 | ORF3_k1_tail_2 | TTGTGTCTCTTTATAGGCCTT | 3´ RACE | S4, S9 |
| K1ORF4 | 5_RACE_O4_K1 | TCCATTAAATCCAGAGTTATTCTTTC | pRKL1-9 verification | S8 |
| K1ORF4 | ORF4-pGKL1_tail | AGAAGCTCTAAATAGTGTTGAAGG | 3´ RACE / pRKL1-4 verification | 2, S1, S4, S9 |
| K1 TIR | K08 | CAACTCTGTATAACAAGTCT | pRKL1-4 verification | 2, S1 |
| K2ORF1 | ORF1-K2_tail_2 | CCTCCATTCTATTTATGGCATAAAAG | 3´ RACE | S4, S9 |
| K2ORF2 | 5RACE_O2_K2 | TTCGTATGTAAATGTTTCCGCA | pRKL2-6/pRKL2-10/pRKL2-11/pRKL2-14 verification | 2, S1, S2 |
| K2ORF2 | K10 | AAGTCCAGATAATACTATAG | 3´ RACE | S4, S9 |
| K2ORF3 | vORF3-k2-forw | AAATCTGGAATCTGCTATGG | ChIP | 3 |
| K2ORF3 | vORF3-k2-rev_2 | GTTCTTTTGTTAGCCGGTATT | ChIP / pRKL2-8/pRKL2-10 verification | 2, 3, S1 |
| K2ORF3 | ORF3-K2_tail_3 | AAGAAGATTGCTTCGAATGTAAAATTG | 3´ RACE / pRKL2-6/pRKL2-10/pRKL2-11/pRKL2-14 verification / sequencing | 2, S1, S2, S4, S9 |
| K2ORF4 | ORF4-K2_tail_3 | CGAATAAATTCAAAATTTCTTGGTGC | 3´ RACE | S4, S9 |
| K2ORF5 | in_ORF5_forw | AGTGGTGAAGAGGAAAAATC | 3´ RACE / pRKL2-3/pRKL2-9/pRKL2-12 verification | 2, S1, S4, S5, S9 |
| K2ORF6 | in_ORF6_forw | TCCCATGGAGTGCATAGAAT | ChIP | 3 |
| K2ORF6 | k2orf6primex | CAACATTTCTAAAATAATCGTTAT | pRKL2-9/pRKL2-12 verification | 2, S1, S5 |
| K2ORF6 | 5RACE_O6_K2 | CTGACCAATTTAATGGTAAATTCC | ChIP / pRKL2-4/pRKL2-9/pRKL2-12 verification / pRKL2-3/pRKL2-4/pRKL2-9/pRKL2-12 sequencing | 1, 2, 3, S1, S5 |
| K2ORF6 | ORF6-K2_tail_2 | CGTTGTTGTGTCAATCATTTAATGCAG | 3´ RACE / pRKL2-5/pRKL2-15 verification | 2, S1, S4, S9 |
| K2ORF7 | 5RACE_O7_K2 | CAAATAGCTCATTTTTGTCATAAGC | pGKL2-derived plasmid detection | S3 |
| K2ORF7 | ORF7-pGKL2_tail | TTTCTCAGAGATAACCCAGAGAAC | pRKL2-5/pRKL2-15 sequencing | 2, S1 |
| K2ORF7 | ORF7-K2_tail_2 | GATTCCACTTTAGAAGACGAAGTAGAAG | 3´ RACE | S4, S9 |
| K2ORF8 | ORF8-pGKL2_tail | CCAATGTTTCATTTGTCGAATACT | 3´ RACE / pGKL2-derived plasmid detection / pRKL2-5/pRKL2-15 verification | 2, S1, S3, S4, S9 |
| K2ORF9 | ORF9-K2_tail_2 | TGTAGTAAAACAAAAAGATATGGCAG | 3´ RACE | S4, S9 |
| K2ORF10 | K27 | AATGGCTAATAAACAGGCAG | 3´ RACE | S4, S9 |
| K2ORF11 | ORF11_K2_tail_2 | TGCCTCTACAAAATAATAAAATAATTCTTG | 3´ RACE / pRKL2-7/pRKL2-13 verification | 2, 3, S1, S4, S9 |

* UCR sequence bearing one point mutation in putative initiator region (INR*)

** UCR sequence bearing two point mutations in putative initiator region (INR**)

° 3′ UTR of K2ORF5 gene bearing mutations in putative Stem loop 2

°° 3′ UTR of K2ORF5 gene bearing rescue mutations in putative Stem loop 2
