# Supplementary material for: Transcription apparatus of the yeast virus-like elements: Architecture, function, and evolutionary origin
Source: PLoS Pathog. 2018 Oct 22;14(10):e1007377. doi: 10.1371/journal.ppat.1007377 (PMC6211774; doi:10.1371/journal.ppat.1007377)
Supplement: S3 Table — (DOCX) [file ppat.1007377.s012.docx]

| **Element to be constructed** | **Primers** | **Template** | **PCR length (bp)** | **Primers for fusion PCR** | **Fusion PCR length (bp)** |
| --- | --- | --- | --- | --- | --- |
| pRKL1-1 | pGKL-kanF  pGKL-kanR | pUG6 | 914 | NA | NA |
| pRKL1-2 | K1O2_prom_del_F  pGKL-kanR | pRKL1-1 | 914 | NA | NA |
| pRKL1-3 | K1O2_prom_del2_F pGKL-kanR | pRKL1-1 | 914 | NA | NA |
| pRKL1-4 | K07  K1_ORF4-HA_R1 | pGKL1 | 805 | K07  K1_ORF4-HA_R2 | 1882 |
|  | K1_ORF4-HA_F2  K1_ORF4-HA_R2 | pcDNA5/FRT/TO | 1104 |  |  |
| pRKL1-5 | pGKL-kanF  kanR2 | pRKL1-1 | 862 | pGKL-kanF  K1_K2O5-UTR_2R | 1084 |
|  | K1_K2O5-UTR_2F  K1_K2O5-UTR_2R | pGKL2 | 249 |  |  |
| pRKL1-6 | pGKL-kanF  kanR2 | pRKL1-1 | 862 | pGKL-kanF  K1_K2O5-UTRm_2R | 1084 |
|  | K1_K2O5-UTR_2F  K1_K2O5-UTRm_2R | pGKL2 | 249 |  |  |
| pRKL1-7 | pGKL-kanF  kanR2 | pRKL1-1 | 862 | pGKL-kanF  K1_K2O5-UTRres_2R | 1072 |
|  | K1_K2O5-UTR_2F  K1_K2O5-UTRres_2R | pGKL2 | 237 |  |  |
| pRKL1-9 | 5RACE_O3_K1_2  K1_UCRmut-O4_R1 | pGKL1 | 516 | 5RACE_O3_K1_2  K1_UCRmut-O4_R2 | 1641 |
|  | K1_HA-ORF3_F2  K1_UCRmut-O4_R2 | pcDNA5/FRT/TO | 1153 |  |  |
| pRKL2-3 | KL_orf6N_Flag1F  KL_orf6N_Flag1R | pRKL1-1 | 957 | KL_orf6N_Flag1F  in_ORF6_rev | 1733 |
|  | KL_orf6N_Flag2F  in_ORF6_rev | pGKL2 | 819 |  |  |
| pRKL2-4 | KL_orf6N_Flag1F  KL_orf6N_EGFP1R | pRKL1-1 | 957 | KL_orf6N_Flag1F  KL_orf6N_EGFP2R | 1731 |
|  | KL_orf6N_EGFP2F  KL_orf6N_EGFP2R | pUG36 | 817 |  |  |
| pRKL2-5  pRKL2-15 | KL_orf7C_Flag1F  KL_orf7C_Flag1R | pGKL2 | 512 | KL_orf7C_Flag1F  KL_orf7C_Flag2R | 1572 |
|  | KL_orf7C_Flag2F  KL_orf7C_Flag2R | pcDNA5/FRT/TO | 1087 |  |  |
| pRKL2-6  pRKL2-10  pRKL2-14 | pGKL_ORF3-HA_F1  pGKL_ORF3-HA_R1 | pcDNA5/FRT/TO | 1115 | pGKL_ORF3-HA_F1  K2_ORF3_for_seq | 1888 |
|  | pGKL_ORF3-HA_F2  K2_ORF3_for_seq | pGKL2 | 803 |  |  |
| pRKL2-7  pRKL2-13 | pGKL_HA-ORF4_F1  pGKL_HA-ORF4_R1 | pcDNA5/FRT/TO | 1117 | pGKL_HA-ORF4_F1  5RACE_O4_K2 | 1424 |
|  | pGKL_HA-ORF4_F2  5RACE_O4_K2 | pGKL2 | 335 |  |  |
| pRKL2-8 | EGFP-ORF4_1F  kanR2 | pRKL1-1 | 897 | EGFP-ORF4_1F  EGFP-ORF4_2R | 1711 |
|  | EGFP-ORF4_2F  EGFP-ORF4_2R | pUG36 | 845 |  |  |
| pRKL2-9  pRKL2-12 | pGKL_HA-ORF6_F1  pGKL_HA-ORF6_R1 | pcDNA5/FRT/TO | 1119 | pGKL_HA-ORF6_F1  in_ORF6_rev | 1911 |
|  | pGKL_HA-ORF6_F2  in_ORF6_rev | pGKL2 | 819 |  |  |
| pRKL2-11 | pGKL2_ORF3-EGFP_F1  KL_orf6C_Flag2F | pRKL1-1 | 932 | pGKL2_ORF3-EGFP_F1  pGKL2_ORF3-EGFP_R2 | 1727 |
|  | pGKL2_ORF3-EGFP_F2  pGKL2_ORF3-EGFP_R2 | pUG36 | 822 |  |  |

NA, not applicable
