# Supplementary material for: Transcription apparatus of the yeast virus-like elements: Architecture, function, and evolutionary origin
Source: PLoS Pathog. 2018 Oct 22;14(10):e1007377. doi: 10.1371/journal.ppat.1007377 (PMC6211774; doi:10.1371/journal.ppat.1007377)
Supplement: S4 Table — (DOCX) [file ppat.1007377.s013.docx]

| **Gene** | **TSS** |
| --- | --- |
| ORF1-pGKL1 | 233 |
| ORF2-pGKL1 | 3227 |
| ORF3-pGKL1 | 7931 C |
| ORF4-pGKL1 | 7920 |
| ORF1-pGKL2 | 228 |
| ORF2-pGKL2 | 3947 C |
| ORF3-pGKL2 | 5726 C |
| ORF3-pGKL2 (long)* | 5818 C |
| ORF4-pGKL2 | 5752 |
| ORF5-pGKL2 | 7400 |
| ORF6-pGKL2 | 7979 |
| ORF7-pGKL2 | 11303 C |
| ORF8-pGKL2 | 11554 C |
| ORF9-pGKL2 | 11523 |
| ORF10-pGKL2 | 12906 |
| ORF11-pGKL2 | 5556 |

The following sequences (with their accession numbers) were used for transcription start site annotations: *K. lactis* pGKL1 (X00762.1), *K. lactis* pGKL2 (X07776.1). C - complementary strand.

* We detected a second K2ORF3 UCS used for transcription *in vivo*, located upstream from the previously reported UCS that produces transcripts with longer 5′ UTR [36].
