# Supplementary material for: Transcription apparatus of the yeast virus-like elements: Architecture, function, and evolutionary origin
Source: PLoS Pathog. 2018 Oct 22;14(10):e1007377. doi: 10.1371/journal.ppat.1007377 (PMC6211774; doi:10.1371/journal.ppat.1007377)
Supplement: Supplementary file 8 [file ppat.1007377.s009.docx]

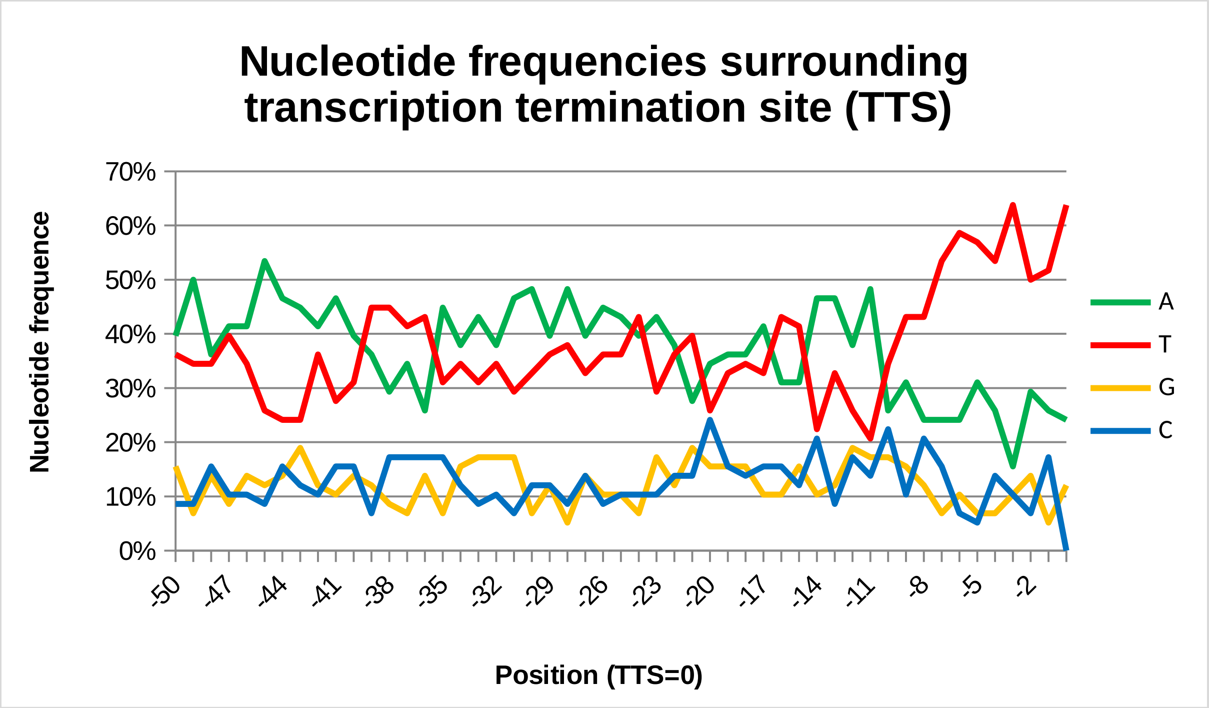

Nucleotide frequencies surrounding transcription termination site (TTS)
Nucleotide frequence
70%
60%
50%
40%
30%
20%
10%
0%
-50
-47
-44
-41
-38
-35
-32
-29
-26
-23
-20
-17
-14
-11
-8
-5
-2
Position (TTS=0)
A
T
G
C
